# Adenosinergic Signaling Alters Natural Killer Cell Functional Responses

**DOI:** 10.3389/fimmu.2018.02533

**Published:** 2018-10-30

**Authors:** Andrea M. Chambers, Jiao Wang, Kyle B. Lupo, Hao Yu, Nadia M. Atallah Lanman, Sandro Matosevic

**Affiliations:** ^1^Department of Industrial and Physical Pharmacy, Purdue University, West Lafayette, IN, United States; ^2^Center for Cancer Research, Purdue University, West Lafayette, IN, United States

**Keywords:** NK cells, adenosine, immunometabolism, immunotherapy, purinergic signaling

## Abstract

Adenosine is a potent immunosuppressive purine metabolite contributing to the pathogenesis of solid tumors. Extracellular adenosine signals on tumor-infiltrating NK cells to inhibit their proliferation, maturation, and cytotoxic function. Cytokine priming imparts upon NK cells distinct activation statuses, which modulate NK anti-tumor immunity and responses to purinergic metabolism. Here, for the first time, we investigated human NK cell responses to adenosinergic signaling in the context of distinct cytokine priming programs. NK cells were shown to be hyper-responsive to adenosine when primed with IL-12 and IL-15 compared to IL-2, exhibiting enhanced IFN-γ expression from CD56^bright^ and CD56^dim^ subsets while modulating the expression of activation marker NKG2D. These responses resulted in signaling that was dependent on mTOR. Adenosine induced upregulation of transcriptional signatures for genes involved in immune responses while downregulating cellular metabolism and other protein synthesis functions that correlate to inhibited oxidative phosphorylation and glycolysis. Overall, our findings show that adenosine acts on specific cellular pathways rather than inducing a broad inhibition of NK cell functions. These responses are dependent on cytokine priming signatures and are important in designing therapeutic interventions that can reprogram NK cell immunometabolism for improved immunotherapies of solid tumors.

## Introduction

Natural killer (NK) cells are powerful effectors of innate immunity. Although immunotherapies utilizing genetically-engineered NK cells are showing promise in clinical settings (1), clinical treatment of solid tumors has lagged behind that of hematologic malignancies (2, 3). Unlike T cells, NK cell function is driven by a balance of activating and inhibitory receptors through which they interact with pathogens by recognizing major histocompatibility complex (MHC) class I molecules on cancer cells (4). The largest group of NK cell receptors includes natural killer group 2 (NKG2), which includes NKG2A, B, C, D, E, F, and H. Except NKG2A and B, all receptors are activating. In pathogenic environments, such as in solid tumors, expression of activating receptors may be impaired, leading to suppression of NK cell anti-tumor immunity (5).

Overall, multiple elements contribute to overall pathogenesis in solid cancers. These elements include hypoxia-driven purinergic signaling, resulting in the accumulation of extracellular adenosine (ADO) (6), a powerful purinergic mediator, signaling on tumor-infiltrating NK cells to negatively regulate their anti-tumor function.

**Figure 1:**
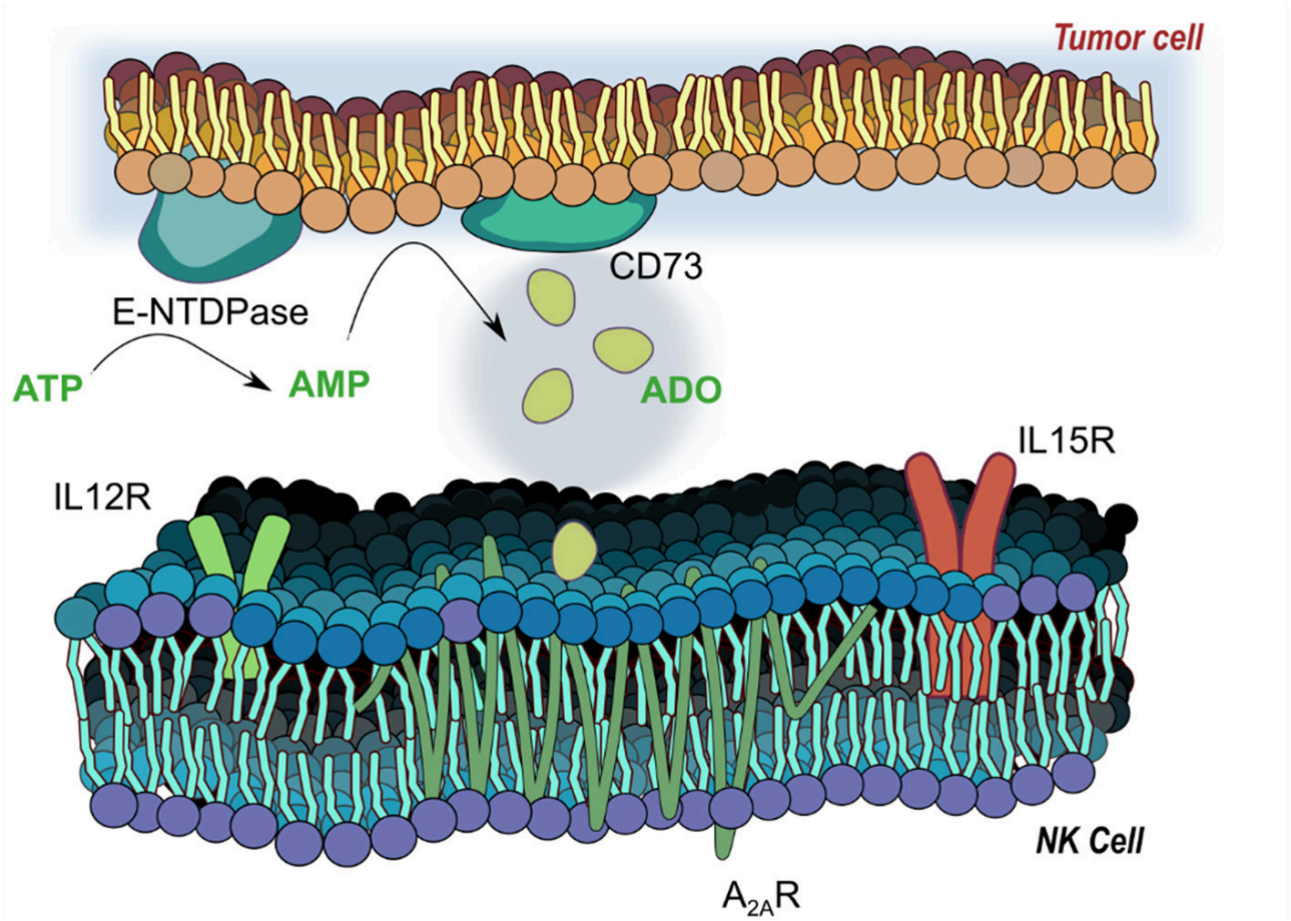
Adenosinergic signaling on natural killer (NK) cells. E-NTDPase (CD39) and 5-ectonucleotidase (CD73) on cancer cells catalyze the dephosphorylation of adenosine triphosphate (ATP) and adenosine monophosphate (AMP) into adenosine (ADO). Extracellular ADO signals via receptors on NK cells—particularly the A_2A_ receptor, a seven-pass transmembrane G protein coupled receptor—to alter functional responses on NK cells, including cytotoxicity, proliferation and activation. Receptors for IL-12 (IL12R) and IL-15 (IL15R) mediate activation of NK cells by the two cytokines, and work to modulate responses to ADO signaling.

The accumulation of extracellular ADO, followed by engagement of G-protein-coupled ADO receptors (A_1_, A_2A_, A_2B_, and A_3_) (7) on tumor-reactive NK cells has emerged a highly immunosuppressive mechanism driving tumor evasion (8). Fast-proliferating cancer cells are characterized by over-utilization of ATP, fueling enzymatic activity of cancer-associated ectoenzymes CD39 and CD73, which catalyze the phosphorylation of AMP to ATP and, finally, extracellular ADO (9). ADO, in turn, interacts with ADO receptors on NK cells, mediating its immunosuppression most strongly via the A_2A_ receptor (A_2A_R) (Figure 1). Elevated expression of CD39 and CD73 (10) on cancer cells was shown to be associated with worse overall survival in solid tumor patients (11, 12). These ectoenzymes were shown to interfere with the trafficking and activities of NK cells into sol id tumor sites via heterologous desensitization of chemokine receptors (13) and reduced proinflammatory cytokines, further promoting cancer development. Extreme dysfunction in purinergic metabolism can additionally manifest in ADO deaminase-severe combined immunodeficiency (ADA-SCID), caused by accumulation of ADO due to ADA loss of function, ultimately resulting in partial or complete lymphopenia (14). As a result, adenosinergic signaling has emerged as a negative regulator of local and systemic anti-tumor response. Debilitating ADO-induced immunosuppression by systemic oxygenation has been shown to weaken ADO-driven tumor protection (15, 16). Similarly, hindrance of ADO accumulation by antibody-mediated blockage of CD39 or CD73, or by A_2A_R antagonism is emerging as a promising checkpoint blockade target. When administered simultaneously, anti-CD73 antibody and A_2A_R inhibitor SCH58261 were shown to decrease the metastatic burden on carcinoma-bearing mice by mobilizing NK cells, CD8^+^ T cells, and interferon-γ (IFN-γ) (17).

The mechanisms through which ADO mediates function of NK cells are varied, though not entirely understood. ADO is known to inhibit TNF-α release from IL-2 stimulated NK cells (18). IL-2 was also found to alleviate ADO-induced immunosuppression of NK cells (19), suggesting cytokine priming to mitigate ADO-induced immunosuppressive effects. ADO's cytotoxic functions have been most extensively linked to engagement of A_2A_R (20), though A_3_ receptor agonism was reported to enhance cytotoxicity of NK cells against tumors in conjunction with IL-12 (21). Moreover, recent insights have emerged linking A_2A_R engagement on NK cells to defective NK cell development, with its antagonism promoting the accumulation of highly cytotoxic CD56^dim^ NK cells (22).

However, not much is known about the direct effects of ADO on NK cell phenotype and function with regards to cytokine priming. While IL-2, IL-12, and IL-15 have been shown to exert powerful effects on the proliferative and cytotoxic capacity of NK cells (23), the presence of ADO in modulating effector responses of cytokine-stimulated NK cells are unknown. Here, we report, for the first time, phenotypic, metabolic, functional and genomic analyses of cytokine-stimulated human NK cells in the setting of ADO signaling. Our results reveal that ADO is inhibitory to NK cell function via dysregulation of specific cellular functions, resulting in downregulation of activating receptor expression and impaired metabolic activity. Functional responses of cytokine-stimulated NK cells are stimulation-dependent, and most pronounced with a combination of IL-12 and IL-15. This knowledge will help direct altered immunometabolic suppression of NK cell function within the context of adoptively-transferred immunotherapies for the treatment of solid tumors.

## Materials and methods

### Reagents and cell lines

Lung adherent epithelial carcinoma A549 cells were obtained from ATCC (ATCC^®;^ CCL-185). A549 cell lines were cultured in DMEM (Gibco, ThermoFisher) with 10% FBS and 1% penicillin/streptomycin (Gibco), and maintained until passage 20 before being discarded. Chronic myelogenous leukemia K562 cells were obtained from ATCC (ATCC^®;^ CCL-243). K562 cell lines were cultured in RPMI-1640 (Life Technologies, ThermoFisher) with 10% FBS and 1% penicillin/streptomycin (Gibco), and maintained until passage 20 before being discarded. Human rhIL-15 and human rhIL-12 were from Gold Biotechnology, human rhIL-2 was from Akron Biotechnology. TGF-β was from ThermoFisher. All other reagents were from Sigma-Aldrich unless otherwise stated.

### Human samples and NK cell isolation

Primary NK cells used in this study were obtained using Purdue University's Institutional Review Board (IRB)-approved consent forms signed by each adult volunteer donor. Blood samples were obtained from twelve healthy adult donors. Natural killer cells were isolated from whole blood by negative selection using the EasySep™ Direct Human NK cell Isolation Kit (StemCell Technologies). The cells were about 80–90% CD3-CD56^+^ when assessed by flow cytometry. Cells were immediately plated at a density of 1 × 10^6^ cells/ml and were cultured in RPMI-1640 (Life Technologies, ThermoFisher) containing 10% FBS, 1% penicillin/streptomycin (Gibco), and accordingly with cytokine, metabolite and small molecule treatment programs as described 37°C, 5% CO_2_ for about 24 h.

### Human NK cell stimulation, ADO treatment and metabolic inhibition

Stimulation programs are as indicated and include priming with IL-2 (200 IU/ml or 400 IU/ml), IL-12 (30 ng/ml) and IL-15 (100 ng/ml), or IL-15 (100 ng/ml). NK cells were also stimulated with or without TGF-β (10 ng/ml) or endogenous ADO (1 mM). Inhibition of the ADO A_2A_ receptor was carried out using SCH58261 (Cayman Chemical) at a concentration of 1 μM. Inhibition of the mTORC1 was carried out using Torin-1 (Cayman Chemical) at a concentration of 1 μM. For the cytotoxicity assays, ADO deaminase inhibitor erythro-9-(2-hydroxy-3-nonyl)adenine (EHNA, Sigma-Aldrich) was used at a concentration of 30 μM.

### Flow cytometry

For flow cytometric analysis, cells were washed with FACS buffer (1X PBS, 5% FBS) and then stained for 30 min at 4°C with select antibodies CD56 (Pe-Cy5.5, Clone: CMSSB/APC, Clone:5.1H11), CD3 (Pe-Cy7, Clone: UCHT1), CD73 (APC, Clone: 82), CD16 (APC, Clone: B73.1), NKp30 (BV711, Clone: p30-15), NKG2D (PE, Clone: 1D11), IFN-γ (Percp-Cy5.5, Clone: B27), pS6 (V450, Clone: N7-548), pSTAT5 (PE, Clone: 47), and DNAM (PE, Clone: DX11) (eBioscience, BD, or BioLegend). Intracellular staining was performed using BD Cytofix/Cytoperm™ (BD Biosciences) with brefeldin A (GolgiPlug) added to cells 4 h before the end of the assay. Sytox™ Green Dead Cell Stain (ThermoFisher) was used to determine cell viability. Gating was performed using fluorescence minus one (FMO) controls and CD3-CD56+, with CD56dim and CD56bright having distinct populations as confirmed by CD16 (Figures S1,S4). Analysis was performed on the BD Fortessa.

### Human NK cell cytotoxicity with target cells

NK cells were isolated and cultured for 20 h with different cytokine stimulations before the addition of A549 or K562 target cells. A549 target cells were labeled with carboxyfluorescein succinimidyl ester (CFSE) for 15 min at 37°C following the 7-AAD/CFSE Cell-Mediated Cytotoxicity Assay Kit (Cayman Chemical) to distinguish between target cells (A549/K562) and effector cells (NK cells). The A459 cells were labeled 4 h before culturing with the NK effector cells to allow for the A549 cells to adhere onto the culture plates, while the K562 cells were set to rest for 1 h in NK cell culture medium (without cytokines or inhibitors) at a density of 1 × 10^6^ cells/ml before culturing with NK cells. Next, the effector cells were incubated with the CFSE labeled A549 or K562 cells target cells at a ratio of E:T of 10:1 in 24 well plates. For the A549 cells, media was removed and replaced with the NK cell culture media and NK cells. The K562 cells were added directly to the NK cell cultures. The NK cells were kept at a density of 1 × 10^6^ cells/ml with the A549 or K562 target cells at a density of 1 × 10^5^ cells/ml. Controls with just A549 cells were also cultured for each test. After coculture for 4 h at 37°C, 5% CO_2_, the supernatant with NK cells was collected, and the A549 cells were washed with 1 X PBS, trypsinized, and then added to the NK cell supernatant. The cultures with K562 were collected directly after 2 or 4 h. The cell mixture was then stained with 7AAD (Cayman Chemical) for 15 min at 4°C. The cells were analyzed on the BD Fortessa instrument (BD biosciences) for both CFSE and 7AAD and data was evaluated using FlowJo software (Tree Star).

### Seahorse metabolic assays

The XFp Extracellular Flux Analyzer (Seahorse Bioscience) was used to measure extracellular acidification rates (ECARs) and oxygen consumption rates (OCRs) for the primary NK cells cultured without cytokines, IL-12 and IL-15, and IL-12 and IL-15 with ADO. XFp Cell Culture Miniplates were coated with 25 μl poly-L-lysine (Sigma) overnight and rinsed with Milli-Q® water before the addition of NK cells (5 × 10^5^ cells/well). After about 30 h of NK cell stimulation, NK cells were resuspended in either XF Cell Mito Stress Test Assay Medium (XF Base Medium supplemented with 2.05 mM L-glutamine, 11 mM glucose, and 1 mM sodium pyruvate) or XF Glycolysis Stress Test Assay Medium (XF Base Medium supplemented with 2.05 mM L-glutamine). Both mediums were adjusted to a pH of 7.4 and filtered before adding to NK cells. Once the NK cells were added to the XFp Cell Culture Mini-plate, the cells were centrifuged at 800 G for 1 min without a break to adhere the NK cells onto the bottom of the miniplates. NK cells were assayed in triplicate and then placed into a non-CO_2_ incubator for 30 min prior to the assays. Stock solutions for each assay were prepared as recommended by the Test Kit guidelines. OCR and ECAR readings were taken over 60–70 min and data was analyzed with the Seahorse XF Stress Test Report Generator.

### RNA isolation and RNAseq library preparation

Freshly isolated, primary NK cells were cultured for 24 h in the presence of IL-12 and IL-15 with ADO as previously described. Cells were harvested and total RNA was isolated from the NK cells using the *mir*Vana™ miRNA Isolation Kit (ThermoFisher) according to manufacturer's instructions, with 4.5 × 10^5^ cells/sample. The samples were then quantified using the Qubit 4 Flurometer (ThermoFisher), and prepared for RNAseq analysis at the Purdue Genomics Core (Purdue University). The dscDNA quality was checked prior to library preparation using an Agilent Bioanalyzer with a High Sensitivity DNA Chip. Samples were prepared for sequencing using the NuGen Universal Plus mRNA-Seq kit (NuGen Technologies Inc.,). Paired-end, 100 bp reads were sequenced on the NovaSeq 6000.

### RNA-seq analysis

Following sequencing on the NovalSeq, Fastx_Toolkit v. 0.0.12.2 quality trimmer (24) was used to trim reads based on quality score. A FastX trimscore of 30, a trim length of 50 were used. Data quality before and after quality trimming was observed using the program FastQC v.0.11.2 (25). Paired-end reads were mapped to the GRCh38.p12 Ensembl reference genome using STAR v. 2.5.4bq (3). Default STAR parameters were used with the exception that library-type was set to “fr-firststrand” and that 1 mismatch was allowed in mapping. After mapping, the htseq-count script in HTSeq v. 0.6.1 (26) was used to count the number of reads mapping to each gene. Biopython v.2.7.3 was used in running HTSeq. HTSeq was run in “intersection-nonempty” mode, the –stranded = reverse option was set, exons were the features used, and “gene_id” was the attribute parameter used. DESeq2 v. 1.20.0 (27) and edgeR v.3.18.1 (28) were used to perform the differential gene expression analysis. The Benjamini–Hochberg method for correcting *p*-values for multiple testing was used. Genes were identified as statistically significant at adjusted *p* < 0.05. A gene set enrichment analysis (GSEA) was used to find sets of genes significantly enriched in control or ADO treated genes. GSEA v. 3. (6) and KEGG, Reactome, GO, and Hallmark gene sets were used in the analysis. We performed GSEA on the pre-ranked dataset, in which genes were ranked using the statistics from DESeq2 and specifically, by the sign of the log_2_ fold-change multiplied by –log_10_(*p*-value). GSEA data has been deposited at GEO under accession number GSE119705.

### Statistical analysis

Prism 7 (GraphPad Software) was used for all statistical analysis with a *p* < 0.05 (^*^) considered to be significant. Ordinary one-way analysis-of-variance tests or the Kruskal–Wallis tests were used for multiple-group comparisons along with the Tukey's multiple comparison test or Dunn's multiple comparison test to compare unpaired sample groups. Unpaired or paired *t*-tests or the Mann–Whitney *U*-test were used for single-data comparisons of independent groups.

## Results

### Priming with IL-2 and IL-15 alter NK cell responses in the presence of adenosine

**Figure 2:**
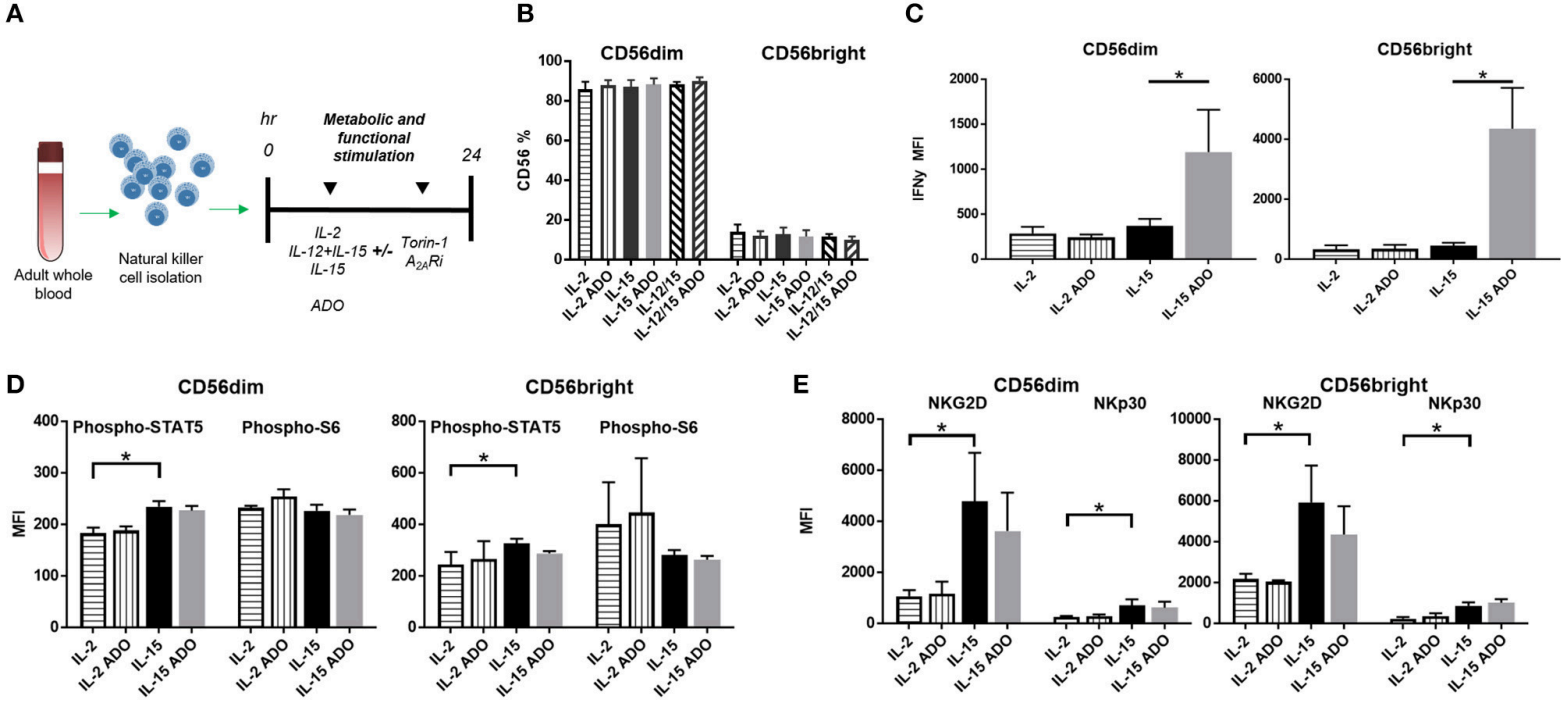
ADO signaling responses by IL-2 and IL-15-stimulated CD56^bright^ and CD56^dim^ NK cells. **(A)** Treatment scheme from blood collection, NK cell isolation and stimulation to analysis. **(B)** Percentage of CD56^+^ NK cells activated with either IL-2, IL-15 or IL-12/IL-15 in the presence or absence of ADO (Unpaired Student *T*-test). In **(C–E)**, human NK cells, sourced from healthy adult donors, were stimulated with IL-2 (200 IU/mL) or IL-15 (100 ng/mL) for 24 h in the presence or absence of ADO (1 mM). **(C)** IFN-γ expression by NK cells in response to ADO and following priming with either IL-2 or IL-15 (Unpaired Student *T*-test or Mann Whitney *U*-test). **(D)** Phosphorylation of STAT5 or s6 in response to ADO on NK cells primed with either IL-2 or IL-15 (Unpaired Student *T*-test or Mann Whitney *U*-test). **(E)** Expression of activating receptors NKG2D and NKp30 on NK cells in response to ADO following priming with either IL-2 or IL-15 (Unpaired Student *T*-test or Mann Whitney *U*-test). Reponses were measured by flow cytometry. *p < 0.05. Data are expressed as means ± SEM.

To determine the effect of ADO on the expression of IFN-γ from cytokine-activated NK cells, we isolated NK cells from fresh adult peripheral blood by negative selection, and stimulated these cells with ADO. Freshly-isolated human PBMC-derived NK cells were gated as CD56^+^/CD3^−^ and further stratified into CD56^dim^ and CD56^bright^ subsets (Figure S1). Overall, ADO treatment did not alter the percentage of CD56^bright^ or CD56^dim^ cells (Figure 2B). IL-2 (200 IU/ml)-stimulated NK cells did not show statistically appreciable alteration in levels of expressed IFN-γ, which remained relatively low and largely comparable to baseline (IL-2-activated cells in the absence of ADO; Figure 2C). When NK cells were stimulated with IL-15 (100 ng/ml), however, we observed a significant increase in the level of expressed IFN-γ in response to ADO (Figure 2B) from both CD56^bright^ and CD56^dim^ NK cells. IFN-γ expression was shown to be highest for CD56^bright^ NK cells. The percentage of IFN-γ^+^ NK cells also increased following ADO treatment (Figures S2A,B).We next investigated changes in the phosphorylation of STAT5 at Y694 (pSTAT5) and ribosomal protein s6 (ps6) at residues S235 and S236 for these activation programs in the presence of the same concentration of ADO. No significant changes in the levels of phosphorylated s6 were observed with either CD56^dim^ or CD56^bright^ NK cells in the response to ADO (Figure 2D). Similar observations were made for the phosphorylation of STAT5 by IL-15-stimulated CD56^dim^ or CD56^bright^ NK cells. IL-15 stimulated CD56^dim^ or CD56^bright^ NK cells both showed a notable increase in the level of phosphorylated STAT5 (pSTAT5) compared to IL-2-stimulated NK cells.

To determine the effect of ADO on the expression of activating NK receptors NKG2D and NKp30, we similarly stimulated NK cells with IL-2 or IL-15 for 24 h in the presence of ADO. ADO induced a decrease in NKG2D from IL-15-stimulated NK cells, though the magnitude of this was sensitive to donor variability (Figure 2E).

### Adenosine alters functional responses and activation markers of IL-12/IL-15-primed NK cells

**Figure 3:**
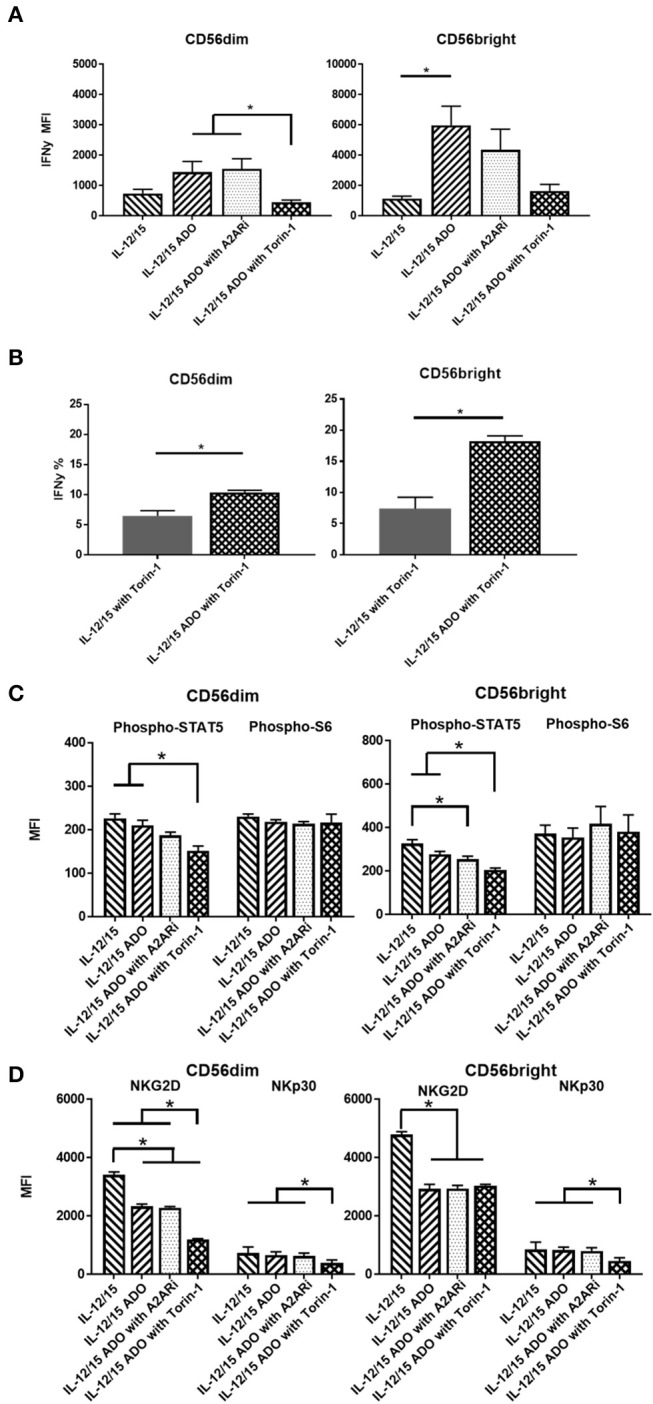
ADO signaling responses by CD56^bright^ and CD56^dim^ NK cells co-stimulated with a combination of IL-12 and IL-15. Human NK cells, sourced from healthy adult donors, were stimulated with a combination of IL-12 (30 ng/mL) and IL-15 (100 ng/mL) for 24 h in the presence or absence of ADO (1 mM). Treatment regime was as illustrated in Figure 2A. **(A)** IFN-γ expression by NK cells in response to ADO and following priming with a combination of IL-12 and L-15, mammalian target of rapamycin (mTOR) inhibitor torin-1 and adenosine A_2A_ receptor inhibitor (A2ARi) SCH58621 (1 μM) (Kruskal–Wallis test with Dunn's multiple comparison). **(B)** Percentage IFN-γ^+^ NK cells following stimulation with IL-12/IL-15 and torin-1 (24 h) in the absence or presence of ADO (Unpaired Student *T*-test). **(C)** Phosphorylation of STAT5 or s6 in response to ADO on NK cells primed with IL-12/IL-15. IL-12/IL-15-co-stimulated human NK cells were also treated, in the presence of ADO, with A_2A_Ri SCH58261 (1 μM) or torin-1 (1 μM). **(D)** Expression of NKG2D and NKp30 on NK cells stimulated as previously, with IL-12/IL-15, torin-1 and A2ARi (ANOVA with Tukey or Kruskal Wallis test with Dunn's multiple comparison). Reponses were measured by flow cytometry. *p < 0.05. Data are expressed as means ± SEM.

An enhanced response to ADO was observed when NK cells were co-stimulated with a combination of IL-12 (30 ng/ml) and IL-15 (100 ng/ml). Under these conditions, CD56^dim^ NK cells yielded an ~2-fold increase in expression of IFN-γ in the presence of ADO. This was comparable to the magnitude of increase observed with the IL-15-stimulated CD56^dim^ subset compared to baseline—stimulated cells without ADO—but resulted in higher overall levels of expressed IFN-γ. Compared to CD56^dim^ cells, IFN-γ expression in the presence of ADO was higher for CD56^bright^ NK cells. Cumulatively, the combination of IL-12 and IL-15 appeared to lead to moderately increased expression of IFN-γ compared to other cytokine stimulation regimens in conjunction with ADO (Figure 3A).

Since we observed increased IFN-γ expression in the presence of ADO with a combination of IL-12 and IL-15, we sought to further investigate this stimulation program. The ADO A_2A_ receptor, present on NK cells, is thought to mediate the cytotoxic response of NK cells in the presence of purine nucleosides (29). To investigate the implication of the A_2A_ receptor on the elevated expression of IFN-γ from ADO and IL-12/IL-15-stimulated NK cells, we treated the cells with small molecule ADO A_2A_ receptor inhibitor (A_2A_Ri) SCH58261 for 24 h. When added to ADO+cytokine stimulated NK cells, A_2A_Ri showed a modest, though not significant, change in expression of IFN-γ (Figure 3A). Though a slight change in A_2A_Ri-mediated reduction in IFN-γ expression was observed, the donor variability likely contributed to the observed results. Because mammalian target of rapamycin (mTOR) was recently implicated in the activation-specific regulation of IFN-γ expression (30), we were also interested in determining the extent of mTOR-mediated metabolic regulation of IFN-γ expression, and the extent to which mTOR is implicated in ADO-mediated regulation of NK cell activation. To determine this, we treated NK cells stimulated with a combination of IL-12/IL-15 with torin-1, a potent ATP-competitive inhibitor of mTOR, and observed a measurable metabolic response. In the presence of torin-1, the expression of IFN-γ from both CD56^bright^ and CD56^dim^ NK cells was significantly dampened, reversing the elevated levels induced by ADO (Figure 3A). An increase in IFN-γ expression was also observed on cytokine-stimulated NK cells treated with torin-1 to which ADO was added (Figure 3B). No changes were observed with expression of NKG2D, NKp30 (Figure S3A), Phospho-STAT5 or Phospho-s6, (Figure S3B) when ADO was added to torin-1 treated cells.

The phosphorylation of s6 remained unaltered in the presence of ADO under IL-12/IL-15 stimulation for CD56^bright^ cells (Figure 3C). This is largely comparable to what was observed with NK cells stimulated with either IL-2 or IL-15 alone. CD56^bright^ NK cells showed a reduction in pSTAT5 in response to ADO and A_2A_Ri. Both CD56^dim^ or CD56^bright^ NK subsets responded to inhibition of mTOR in the presence of ADO by inducing a decrease in pSTAT5.

ADO remained suppressive to the expression of activating receptor NKG2D on NK cells stimulated with IL-12 and IL-15, while NKp30 was not affected by ADO (Figure 3D). A reduction in expression of NKG2D was observed in the presence of ADO on both CD56^bright^ and CD56^dim^ NK cells. Inhibiting mTOR further decreased the expression of NKG2D on CD56^dim^ NK cells. On the other hand, torin-1 inhibition of mTOR also decreased NKp30 expression on both CD56^bright^ and CD56^dim^ NK cells. Similarly to its limited effect on IFN-γ and STAT5/s6 phosphorylation, A_2A_Ri did not exert a measurable change in expression of either activating receptor. Individual donor data shows moderate variability in IFN-γ expression following ADO treatment (Figure S5).

### Human CD16^+^/CD56^+^ NK cells do not express significant CD73

**Figure 4:**
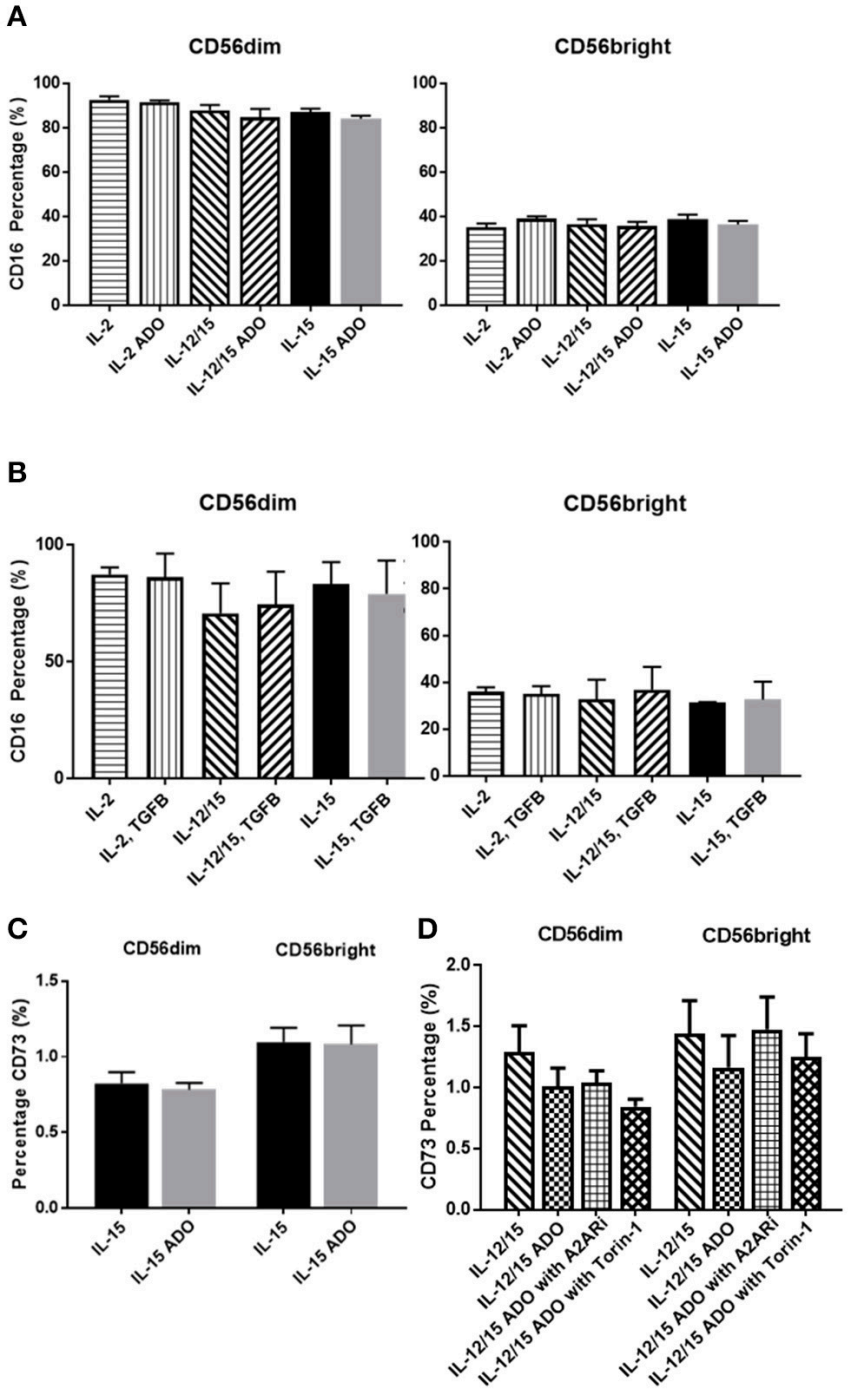
Phenotypic signatures of CD56^bright^ and CD56^dim^ NK cells in response to ADO and TGF-β signaling. Human NK cells, sourced from healthy adult donors, were stimulated with either IL-2 (200 IU/mL), IL-15 (100 ng/mL) or a combination of IL-12 (30 ng/mL) and IL-15 (100 ng/mL) for 24 h in the presence of ADO (1 mM) or TGF-β (10 ng/mL). **(A)** Percentage of CD16^+^ CD56^dim^ and CD56^bright^ NK cells primed with IL-2, IL-15 or a combination of IL-12 and IL-15 in the presence or absence of ADO. **(B)** Percentage of CD16^+^ CD56^dim^ and CD56^bright^ NK cells primed with IL-2, IL-15 or a combination of IL-12 and IL-15 in the presence or absence of TGF-β (Unpaired Student *T*-test). **(C)** CD73 expression on CD56^bright^ and CD56^dim^ NK cells primed with either IL-2 or IL-15 in the absence or presence of ADO. **(D)** CD73 expression on CD56^bright^ and CD56^dim^ NK cells primed with a combination of IL-12 and IL-15 in the absence or presence of ADO, ADO A_2A_ receptor inhibitor SCH58261 (1 μM) or mTOR inhibitor torin-1 (1 μM) (Ordinary one-way ANOVA with Tukey's multiple comparison test). Data are expressed as means ± SEM.

The amounts of CD16^+^ NK cells were consistent throughout all treatment programs and in response to either ADO (Figure 4A) or TGF-β (Figure 4B), with the CD56^dim^ subset being significantly more CD16^+^.

Though CD73, expressed on cancer cells, is primarily involved in aenosinergic metabolism by catalyzing the last step of the conversion of ATP to ADO, NK cells are known to express very low levels of CD73. It was recently suggested that NK cells have the ability to enhance CD73 expression under certain conditions *in vivo*, such as exposure to mesenchymal stem cells (31). We confirmed that expression of CD73 on human NK cells isolated from peripheral blood and stimulated with either IL-15 alone or a combination of IL-12 and IL-15 was low, observable on <2% of cells (Figure 4C). CD73 expression was comparable across both CD56^dim^ and CD56^bright^ populations. For IL-12/IL-15-co-stimulated NK cells, CD73 expression remained unaltered when NK cells were treated with ADO or A_2A_Ri. Similarly, inhibition of mTOR resulted in unaltered levels of CD73, except for CD56^dim^ cells, where a slight reduction in CD73 expression was observed, though not significant when donor variability was taken into account (Figure 4D). Because the overall level of CD73 expression is low across the entire NK cell population, this change is unlikely to result in significant functional alterations.

**Figure 5:**
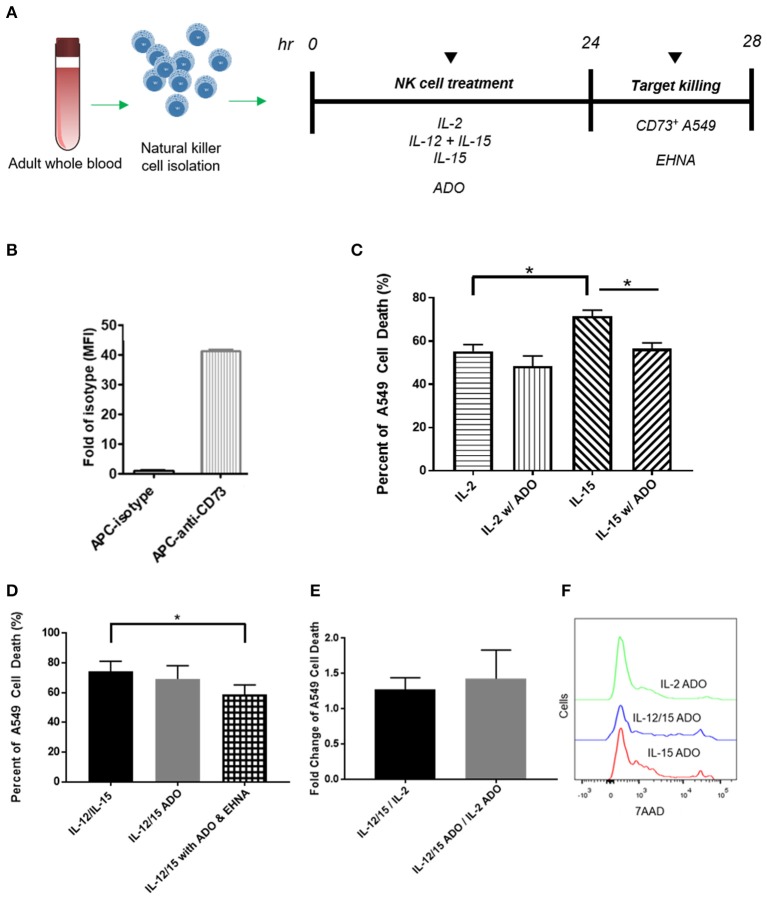
Cytotoxicity of human NK cells against CD73^+^ cancer cells in the presence of ADO. **(A)** Cytotoxicity assay treatment scheme. Freshly-isolated human NK cells were primed with either IL-2 (400 IU/mL), IL-15 (100 ng/mL) or a combination of IL-12 (30 ng/mL) and IL-15 (100 ng/mL) for 24 h in the presence of ADO (1 mM) and in the presence or absence of ADO deaminase inhibitor erythro-9-(2-hydroxy-3-nonyl)adenine (EHNA, 30 μM). 24 h later, NK cells were challenged with A549 cells and their killing ability was measured by flow cytometry. **(B)** Expression of CD73 on A549 lung carcinoma cells. **(C)** Cytolysis of A549 cells mediated by human NK cells primed with either IL-2 or IL-15 in the absence of presence of ADO. **(D)** Cytolysis of A549 cells mediated by human NK cells primed with a combination of IL-12 and IL-15 in the absence of presence of ADO and EHNA. **(E)** A549 cell death mediated by human NK cells expressed as fold-change between NK cells stimulated with IL-12/IL-15 and IL-2 in the presence or absence of ADO. **(F)** Representative flow cytometry traces of A549 cytolysis due to NK cells stimulated with each of three separate cytokine groups in the presence of ADO as measured with 7-AAD staining (Panels **B** and **C** use Unpaired Student *T*-test). *p < 0.05. Data are expressed as means ± SEM.

Like ADO, TGF-β is considered a powerful immunosuppressant. Evidence for this is abundant—TGF-β has been extensively studied and was recently described to inhibit functions of NK cells by repressing the mTOR pathway (32). Apart from downregulating NKG2D expression on NK cells (Figure S6), TGF-β was also shown to induce CD73 expression on cancer cells and endow these cells with the capacity to enhance production of ADO from AMP (33). We found that, while A549 cells express CD73 (Figure 5B), TGF-β was capable of further inducing higher expression of CD73 (Figure S7).

### Adenosine metabolism alters human NK cell cytotoxicity

Our results indicated that human NK cells, when stimulated with a combination of IL-12 and IL-15, displayed elevated expression of IFN-γ in response to ADO. To determine whether the elevated expression of IFN-γ results in either a corresponding increase in NK cell cytotoxicity or an alteration thereof, we sought to determine these cells' cytolytic function against CD73-expressing cancer cells in the presence of ADO. Freshly-isolated human NK cells were pre-activated with either IL-2 alone, IL-15 alone, or a combination of IL-12 and IL-15 in the presence of absence of ADO prior to being challenged with CD73^+^ A549 cells (Figure 5A). NK-mediated killing of A549 cells was quantified by flow cytometry. While these cancer cells express high amounts of CD73 (Figure 5B), we added ADO to pre-treat NK cells in the same way we had done when measuring expression of IFN-γ, as well as to ensure ADO was present at saturating levels during killing. This allowed us to isolate observations as being primarily due to the excess extracellular ADO present in culture. We also measured the expression of MHC class I molecules on these cancer cells, and found they express high amounts of human leukocyte antigens (HLAs) (Figure S9A).

The addition of a high concentration of exogenous ADO did not lead to a significant reduction in cytolysis against A549 cancer cells by cytokine-stimulated NK cells when primed with either IL-2 or a combination of IL-12 and IL-15 (Figures 5C,D). NK cells stimulated with IL-15 showed a slight reduction in killing ability when ADO was added. We did observe that, in the absence of ADO, IL-12/IL-15-co-stimulated NK cells yielded the greatest percentage of A549 killing compared to NK cells stimulated with either IL-2 or IL-15 alone. NK cytotoxicity was also sensitive, as expected, to donor variability. While this could be correlated to our earlier observation that IL-12/IL-15 co-stimulated NK cells show enhanced IFN-γ expression, elevated IFN-γ expression in the presence of ADO did not translate to a proportional increase in NK cell cytotoxicity *in vitro*.

These results led us to question whether a secondary pathway within the extracellular adenosinergic cascade is implicated in regulating the metabolism of ADO to ultimately affect its immunosuppressive function. In order to investigate this, we treated NK cells, primed with IL-12 and IL-15 in the presence of endogenous ADO, with erythro-9-(2-hydroxy-3-nonyl)adenine (EHNA), a potent ADO deaminase inhibitor, at a concentration of 30 and 100 μM, for 24 h. When we treated cytokine-primed NK cells with EHNA, we observed a reduction in cytolysis of A549 cells even when excess endogenous ADO was present (Figure 5D). This was true across multiple donors and at EHNA concentrations of 30 and 100 μM (Figure S8). This led us to speculate that extracellular ADO metabolism may have the ability to regulate NK cell functional responses, and controlling this metabolic cascade may contain potentially important implications for anti-cancer therapy. IL-12/IL-15 priming yielded increased cytolysis against target cells compared to priming with IL-2 alone (Figures 5E,F). One caveat to these analyses is that the high number of NK cells required for cytolysis, alongside the activation-specific and expansion-independent nature of the experiments, precluded independent analysis of CD56^bright^ and CD56^dim^ subsets, and these results reflect responses of the overall NK population. NK cells were also challenged to lyse K562 cells, which are known to express high amounts of NKG2D ligand, NK cells activated with IL-12/IL-15 demonstrated extremely high cytolysis (Figure S9B) in the presence or absence of ADO.

### ADO impairs NK cells' oxidative phosphorylation and glycolytic capacity

**Figure 6:**
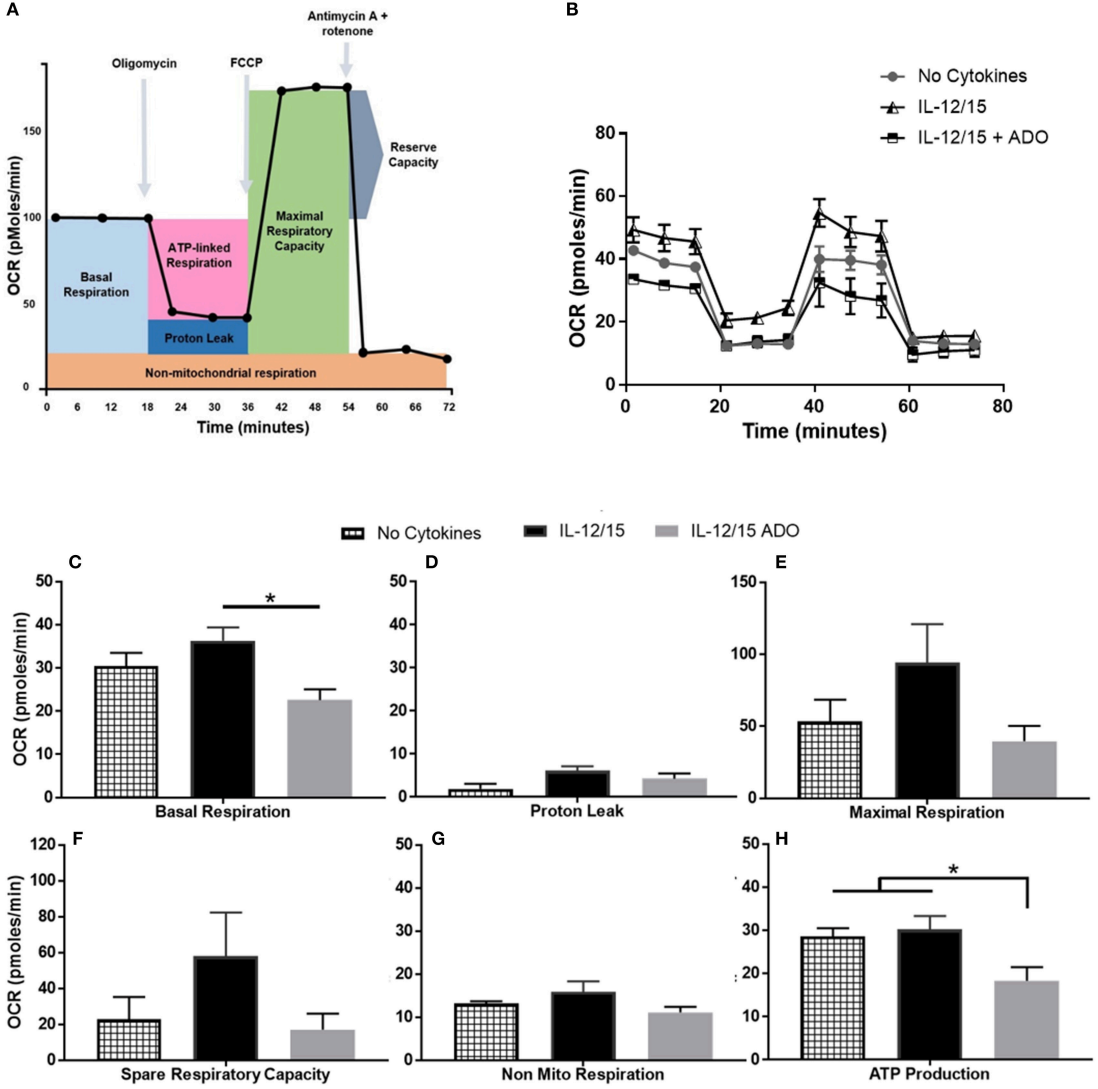
Effects of energy substrates on mitochondrial respiration of IL-12/IL-15 co-stimulated NK cells in the presence of ADO. **(A)** Schematic of mitochondrial stress test, **(B)** Oxygen consumption rate (OCR) and **(C–H)** individual plots for basal respiration, proton leak, maximal respiratory capacity, reserve respiratory capacity, non-mitochondrial respiration and ATP-linked respiration. Freshly-isolated human NK cells were incubated for 24 h in the presence or absence of IL-12/IL-15 or ADO. Oxygen consumption rate was measured in XF base medium supplemented with glutamine, glucose and sodium pyruvate followed by the sequential addition of oligomycin (0.5 μM), FCCP (1 μM), and rotenone + antimycin A (1 μM), as indicated. Each data point represents an OCR measurement (Kruskal-Wallis test with Dunn's multiple comparison test). Data are expressed as means ± SEM, *n* = 3 independent experiments. *p < 0.05.

**Figure 7:**
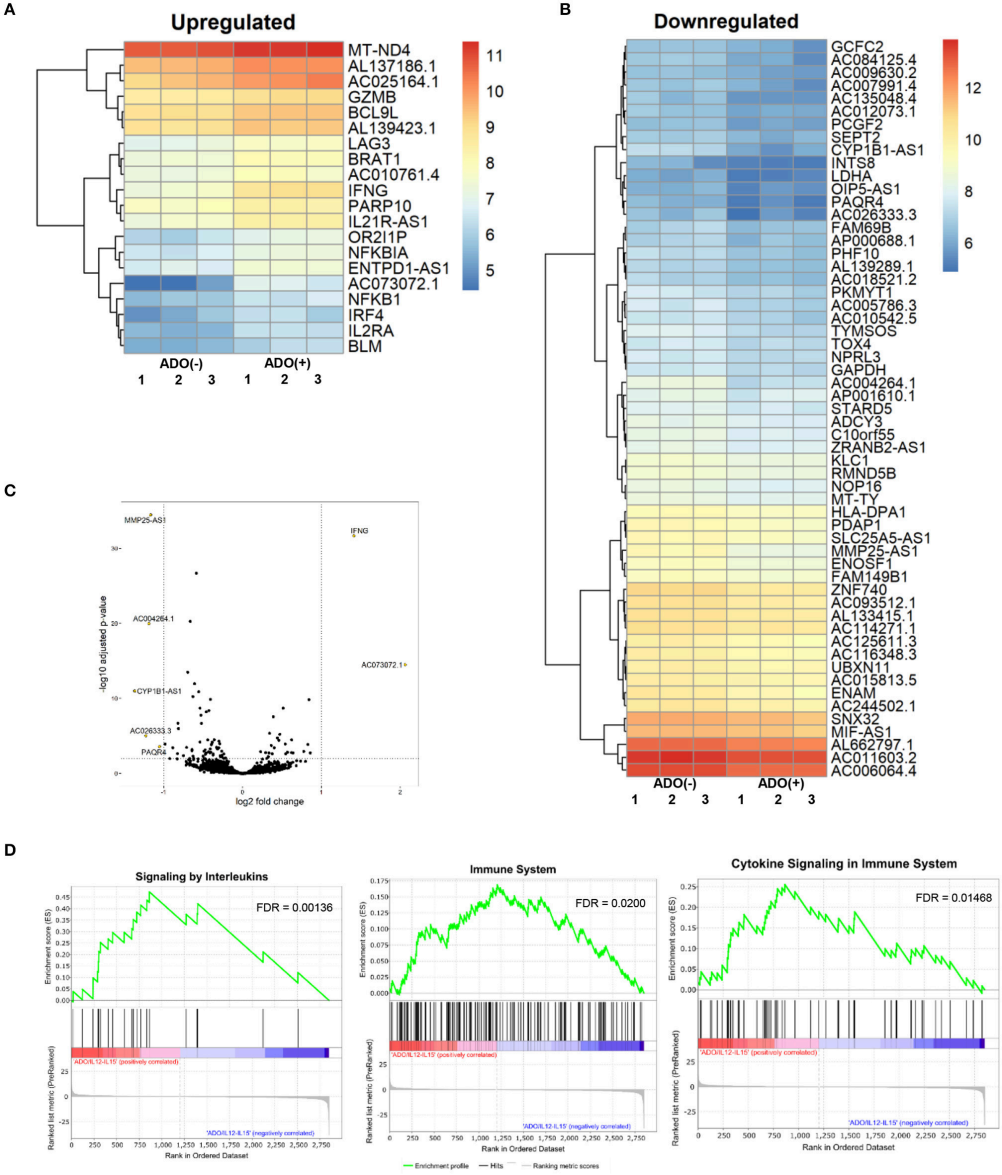
RNAseq analysis of human NK cell responses to ADO treatment and co-stimulation with IL-12 and IL-15. **(A)** Heatmap of differentially-expressed upregulated genes in response to ADO treatment of IL-12/IL-15-activated human NK cells. Differentially-expressed genes were identified through DEseq2 and edgeR database analysis. **(B)** Differentially-downregulated genes in response to ADO treatment of IL-12/IL-15-activated human NK cells based on DEseq2 and edgeR database analysis. **(C)** Volcano plot of gene expression changes in IL-12/IL-15 co-stimulated NK cells in response to ADO. **(D)** GSEA analysis of three most heavily enriched gene sets based on positive transcriptional associations for IL-12/IL-15 co-stimulated NK cells in the presence of ADO.

Though ADO was shown to have a profound effect on NK cells across multiple cytokine stimulation regimens, co-stimulation with IL-12 and IL-15 yielded the most noticeable responses. In line with this, we wanted to focus on understanding the effect of ADO on the metabolic requirements of IL-12/IL-15 co-stimulated NK cells. While the metabolic requirements of IL-12/IL-15 co-stimulated NK cells were previously described (34), we sought to establish the metabolic response in the presence of saturating concentrations of ADO. To do that, we measured the rate of mitochondrial oxygen consumption (OCR), which represents oxidative phosphorylation levels (Figure 6), and the ECAR, as a measure of glycolysis (Figure S10). ADO induced inhibition of oxidative phosphorylation capacity of NK cells. Although IL-12/IL-15 co-stimulation enhanced NK cells' oxidative phosphorylation activities, we observed differences in rates of basal, maximal, or ATP-linked respiration, spare respiratory capacity or non-mitochondrial respiration and proton leak (Figures 6C–H) when ADO was present. Elevated ECAR levels were maintained by IL-12/IL-15 co-stimulation, while ADO induced a profound inhibition in glycolysis and glycolytic capacity of IL-12/IL-15-stimulated NK cells (Figure S10). The potential contribution of key enzymes to ADO-dependent inhibition of glycolysis and oxidative phosphorylation were inferred by altered transcriptional signatures due to ADO signaling. Downregulation of *LDHA* and *GAPDH* genes was observed in ADO-treated samples (Figure 7B). *GAPDH* encodes glyceraldehyde 3-phosphate dehydrogenase, which is involved in the sixth step of glycolysis and ultimately the cellular production of two molecules of ATP. Lactate dehydrogenase A (the M subunit of lactate dehydrogenase), encoded by *LDHA*, converts pyruvate into lactate yielding the protons measured as ECAR.

### ADO alters transcriptional signatures of human NK cells

We performed transcriptional gene expression analysis to gain insight into the impact of ADO stimulation of NK cell biology at the transcriptional level, for cells primed with a combination of IL-12 and IL-15. 77 differentially-expressed genes were identified (Table S1) between DESeq2 and edgeR analyses for the ADO treatment groups. Among them, 20 (25.9%) were up-regulated (Figure 7A), of which 15 could be annotated (Table 1). ADO stimulation was positively associated with expression of genes involved in cytokine and immune signaling. Notably, significant up-regulation of the *IFNG* gene was observed, which correlated with enhanced IFN-γ expression measured by flow cytometry. This was accompanied by differential expression of the interferon regulatory factor 4 (*IRF4*) gene. ADO also induced up-regulation of both *NFKBIA* and *NFKB1* genes, both of which are implicated in the control of the NFκB protein. The upregulation of WNT signaling gene B-cell CLL/lymphoma 9-like (BLC9L), which has been associated with pathology and progression of a number of cancers (35), was also induced in ADO-treated samples. Metabolically, ADO also caused the upregulation of *MT-ND4*, which encodes NADH dehydrogenase 4, a protein that is required for oxidative phosphorylation. In addition, positive correlation was observed with the enhanced differential expression of PARP10, a gene that encodes poly [ADP-ribose] polymerase 10, a mono-ADP-ribosyltransferase that has recently emerged as an oncogene which suppresses tumor metastasis through association with Aurora 1 (36). Of interest is the upregulation of *LAG3* gene, which encodes lymphocyte activation gene-3, a protein that has emerged as an immune regulator and an checkpoint receptor on immune cells. Its role in the function of NK cells is not fully clear yet, or is its interaction with ADO (37).

**Table 1:** Differentially-expressed genes on NK cells in response to ADO.

**UP gene**	**Log2FC**	***P*-value**	**DOWN gene**	**Log2FC**	***P*-value**
*IFNG*	1.41	2.13E-32	*CYP1B1-AS1*	−1.36881	1.09E-12
*IL2RA*	0.86	0.001819	*MMP25-AS1*	−1.16648	3.57E-21
*IL21R-AS1*	0.84	1.44E-10	*PAQR4*	−1.05382	0.000254
*OR2I1P*	0.83	0.000142	*INTS8*	−0.9242	0.008671
*IRF4*	0.80	0.02539	*LDHA*	−0.83027	0.031923
*BLM*	0.74	0.022601	*TYMSOS*	−0.82023	1.64E-06
*NFKB1*	0.69	0.019629	*PCGF2*	−0.74988	0.014609
*NFKBIA*	0.64	0.00182	*OIP5-AS1*	−0.71726	0.068605
*BRAT1*	0.62	3.3E-05	*PHF10*	−0.71006	0.001572
*ENTPD1-AS1*	0.62	0.00165	*ENAM*	−0.69667	4.39E-08
*LAG3*	0.57	0.001477	*GCFC2*	−0.68503	0.02925
*PARP10*	0.54	0.000119	*ZNF740*	−0.66312	8.74E-08
*GZMB*	0.46	3.73E-05	*PKMYT1*	−0.64201	0.002395
*MT-ND4*	0.39	2.75E-08	*ENOSF1*	−0.63064	1.3E-05
*BCL9L*	0.44	1.83E-05	*SEPT2*	−0.62267	0.040442

*Top 15 annotated upregulated and downregulated genes on IL-12 and IL-15 co-stimulated human NK cells in response to ADO as determined via DESeq2 and presented as log2(Fold Change). P-value represents the adjusted P-value*.

We also observed 57 (74.02%) downregulated genes (Figure 7B; Table S1). Among them is *ADCY3*, which has recently been linked to the increased cytokine production that accompanies trained immunity (38). Also downregulated were glycolytic gene *GAPDH* and *LDHA*, hinting at altered metabolism of cells in the presence of ADO, as well as cell cycle control genes including *PKMYT1* and *KLC1*. Collectively, ADO treatment suggested impairment in functions that support NK cellular metabolism and division, which would ultimately lead to inhibition of effector functions. A volcano plot identified *IFNG* as the most highly upregulated gene following ADO and cytokine stimulation (Figure 7C).

A GSEA was performed on all genes to determine the top gene sets, pathways, and gene ontology (GO) terms associated with ADO treatment of IL-12/IL-15-activated NK cells (Figure 7C; Table S2). Gene ontologies were found to be heavily enriched in genes involved in signaling by interleukins and cytokines, as expected following IL-12/IL-15 and ADO stimulation. Other enriched gene ontologies included interferon gamma signaling and antigen processing cross-presentation (Figure 7D), as well as protein translation (Figure S11). This might infer that regulation of activating/inhibitory receptors on NK cells is in response to ADO. Regarding gene sets that were associated with downregulated differentially-expressed genes in response to ADO treatment, gene ontologies represented include protein translation, translational regulation, cell cycle and metabolism of proteins. ADO treatment did not produce any significant pathway alteration. Dependences on ADO for biological pathways are difficult to infer due to the relative limited number of differentially-expressed genes.

## Discussion

The tumor microenvironment's hypoxic cores lead to immunosuppression via dysregulation of natural killer (NK) cells (39) through mechanisms that modulate adenosinergic signaling. Hypoxia in the tumor microenvironment is a powerful mediator of the activities of the ecto-nucleoside triphosphate diphosphohydrolase CD39 and ecto-5′-nucleotidase CD73 (40–42) These enzymes constitute a catalytic cascade which leads to the generation of ADO (43, 44). Though extracellular ADO is present at low micromolar concentrations (45), in response to hypoxia, such as inside cores of solid tumors, ADO can reach hundreds of micromolar or even millimolar concentrations (46). Adenosinergic immunosuppression of infiltrating NK cells signals through four purinergic ADO receptors to inhibit the maturation of NK cells (22), the accumulation of cytotoxic CD56^dim^ cells at tumor sites (22) and NK effector function (20). Among these, the A_2A_ receptor has been most widely studied (47). Unlike other immune cells, NK cells can tolerate elevated concentrations of ADO, though they undergo profound alterations in functional response in such environments. Many of these functional changes had not been characterized in terms of cytokine-primed NK activation, phenotype, and function, and under conditions that induce inhibition of NK cell function. The high ADO concentrations used in this study did not lead to cell death or observable loss of viability during the treatment period. Due to the metabolic instability of ADO, the use of ADO is frequently superseded by the use of its metabolically-stable analog 2-chloroadenosine (CADO). Because CADO might be signaling divergently from ADO, its use might have different immunological implications (48).

Here, for the first time, we showed that adenosinergic signaling leads to significant and distinct reprogramming of functional and anti-tumor responses from human NK cells following specific cytokine priming programs. Generally, cytokine priming attenuated immunosuppression induced by the presence of ADO. This was not surprising, given that IL-2 was shown to dampen inhibition of NK cells due to ADO signaling (18). These responses, however, vary dependent on cytokine activation. In the setting of ADO signaling, IL-2-primed human NK cells remain inferior producers of IFN-γ from either CD56^dim^ or CD56^bright^ subsets, compared to IL-15 alone or a combination of IL-12 and IL-15. Individual contributions from both IL-12 and IL-15 signaling contribute to the overall increase in IFN-γ expression in the presence of ADO, likely through engagement of signaling other than the phosphorylation of STAT5, which is engaged mostly due to IL-15 alone. IL-12, a potent inducer of IFN-γ expression in NK cells signals through STAT4 (49), and could be amplifying the response induced by IL-15. Enhancement of IFN-γ expression in the presence of torin-1 following the addition of ADO compared to torin-1 alone indicates that ADO-mediated IFN-γ activation is sensitive to mTOR signaling but may also be acting independently of it. Following prolonged IL-12/IL-15 stimulation, the mechanism of ADO-mediated NK cell responses could be IL-15 signaling-independent, suggesting an altered activation state (50). Moreover, we showed that ADO affects metabolic functions of NK cells, so downstream signaling may be implicating other pathways tham those involving STAT5/s6. It is not unreasonable to assume that ADO might be activating pathways similar to impaired IL-15 signaling which imply an mTOR and IL-15-independent activation phase, with a shift in metabolism to an activating receptor dependency to maintain NK effector functions (50). Our prolonged cytokine stimulation regimen with a combination of IL-12/IL-15, combined with donor variability, could be dampening Phospho-s6 expression (51), which is otherwise expected to be engaged in response to mTOR signaling.

Although we measured statistically-significant increases of certain NK cell responses to IL-12/IL-15 stimulation compared to either IL-15 or IL-2 alone, the extent of the increase of IL-12/IL-15-mediated responses over stimulation with IL-15 alone was not dramatic, and speaks to the potent effect that IL-15 has on NK cells. Elevated IFN-γ expression, particularly from CD56^bright^ NK cells, is consistent with their profile as being more potent IFN-γ producers, while having an elevated expression of metabolic markers (34). Mechanistically, accumulation of cAMP promotes ADO inhibition of IL-2-induced NK cell proliferation (52). RNAseq data confirmed that ADO induces activation of cytokine signaling responses by upregulating genes involved in immune responses induced by IL-12 and IL-15 priming events, hinting at implications potentially relevant to the use of cytokine-activated NK cells in adoptive cell therapy of solid tumors. Though ADO induced upregulation of *NKFBIA* and *NFKB1* gene expression, and given that ADO causes a cAMP-dependent suppression of NF-κB activity that requires nuclear localization (53), it is still possible that induction of NF-κB is stunted due to ADO inhibition of protein synthesis and metabolism. This is corroborated by altered transcriptional signatures of genes involved in protein translation and downregulated gene sets, based on GSEA analysis, within the protein translation machinery.

As a powerful inducer of ADO production, hypoxia is known to alter expression of activating receptors NKG2D and NKp30 and intracellular perforin and granzyme B (54). Their expression can be restored by priming NK cells with IL-2 (19). As observed, at high concentrations, ADO could be acting independently of hypoxia to induce the downregulation of the NKG2D receptor on NK cells through mechanisms that are independent of A_2A_ receptor signaling but are sensitive to mTOR signaling. Alongside ADO, hypoxic conditions induce the release of TGF-β, a tumor-derived cytokine which exerts a profound immunosuppressive effect on NK cells (55) and likely enhances the inhibition of NK cell function caused by extracellular ADO, collectively creating a niche promoting tumor progression.

The observed limited and variable effects of A2ARi blockade could be due to overactivation of NK cells—it was shown that the effects of SCH58261 dampened at higher cytokine concentrations (56)—donor variability or signaling independent of A_2A_.

Elevated IFN-γ expression induced by IL-12 and IL-15-primed NK cells in response to ADO did not translate to a proportionally-enhanced lytic activity against CD73^+^ tumor targets. It is possible that ADO might be interfering with granule exocytosis from NK cells (57), thus limiting lysis of CD73^+^ cancer cells despite enhanced expression of intracellular IFN-γ. RNAseq data hinted at stunted protein synthetic and transport machinery, suggesting that metabolic functions might be inhibiting NK cytolysis despite enhanced cytokine-induced immune responses to ADO. At the same time, metabolism of ADO might be contributing to the modulation of its effects. Extracellularly, newly-produced ADO is deaminated to the nucleoside inosine. This conversion is catalyzed by ADO deaminase (ADA), an amino hydrolase (58). Indeed, inhibiting ADO deaminase activity with a potent ADO deaminase inhibitor, EHNA, inhibited the cytolytic ability of IL-12/IL-15 co-stimulated NK cells. In our experience, the overall effect of inhibiting ADO deaminase was subtle, however, and increased when higher concentrations of EHNA were used (Figure S8). NK cells' cytotoxic activity is mediated by the level of expression of MHC molecules—HLA-A, HLA-B, and HLA-C—and KIR receptors on cancer cells. A549 cells express high levels of HLA ligands (Figure S9A), possibly limiting cytotoxic activity of NK cells. Cytotoxicity of NK cells toward targets with high known amounts of NKG2D ligands (such as K562 cells) following IL-12/IL-15 activation, induced profound killing of these target cells during shorter timeframes (Figure S9B). Overactivation of NK cells likely contributed to their superior ability to lyse targets, and caused rapid onset of K562 cell death, somewhat skewing lysis data due to a rapidly accumulating large number of dead cells.

Recently, inhibition of ADO deaminase with EHNA has been proposed as a potential target for malignant plural mesothelioma owing to its role in the regulation of the apoptotic effects of ADO (59), and PEGylated ADO deaminase inhibitors as potential anti-cancer targets are being developed (60). When culturing the NK cells with the A549 or K562 cells, the EHNA could have facilitated additional target cell death due to the ability of the EHNA to prevent ADO breakdown and increase the apoptotic effects of ADO. However, extracellular adenosine has an adverse effect on NK cell metabolic activity; therefore, the use of EHNA as a potential target may not be beneficial and may reduce NK cell activity and decrease NK cytolytic effects.

We found that NK cells alter their functional responses to adenosinergic signaling via mechanisms that are sensitive to specific cytokine activation programs, while collectively requiring mTOR signaling. Glycolysis and oxidative phosphorylation are essential for purinergic metabolism, and ADO's inhibitory effect on NK cells' mitochondrial respiration is the first direct demonstration of its involvement in the suppression of NK cell metabolic activity. Hyper-responsiveness to co-stimulation with IL-12 and IL-15 correlated to altered gene expression signatures and cytotoxic responses, with our results suggesting the use of this cytokine combination as being preferential to the use of IL-2, though the individual contributions of IL-12 and IL-15 may require further investigation. Donor variability is an important consideration because distinct subpopulations obtained from different donors respond differently to *ex vivo* treatment. Overall, these results demonstrate that ADO, even at high concentrations, acts on specific cellular pathways rather than causing general NK cell inhibition. These findings have important implications for redirecting the function of NK cells when targeting solid tumors.

## Ethics statement

Written informed consent was obtained from all subjects involved in the study. All procedures performed in studies involving human participants were approved by Purdue University's Institutional Review Board (IRB). All institutional safety and biosecurity procedures were adhered to.

## Author contributions

AC and SM designed the experiments. AC, JW, and KL performed the experiments. HY and NAL carried out RNAseq analysis. AC and SM analyzed the data and wrote the manuscript.

### Conflict of interest statement

The authors declare that the research was conducted in the absence of any commercial or financial relationships that could be construed as a potential conflict of interest.
