# Supplementary material for: Adenosinergic Signaling Alters Natural Killer Cell Functional Responses
Source: Front Immunol. 2018 Oct 30;9:2533. doi: 10.3389/fimmu.2018.02533 (PMC6218627; doi:10.3389/fimmu.2018.02533)
Supplement: Supplementary file 1 [file Data_Sheet_1.docx]

Supplementary Material

**Adenosinergic signaling alters natural killer cell functional responses**

**Andrea Chambers,^1^ Jiao Wang,^1^ Kyle Lupo,^1^ Hao Yu,^2^ Nadia Atallah,^2^ Sandro Matosevic^1,2,*^**

^1^ Department of Industrial and Physical Pharmacy, Purdue University, West Lafayette IN, USA

^2^ Center for Cancer Research, Purdue University, West Lafayette, IN USA

*** Correspondence:** sandro@purdue.edu

**
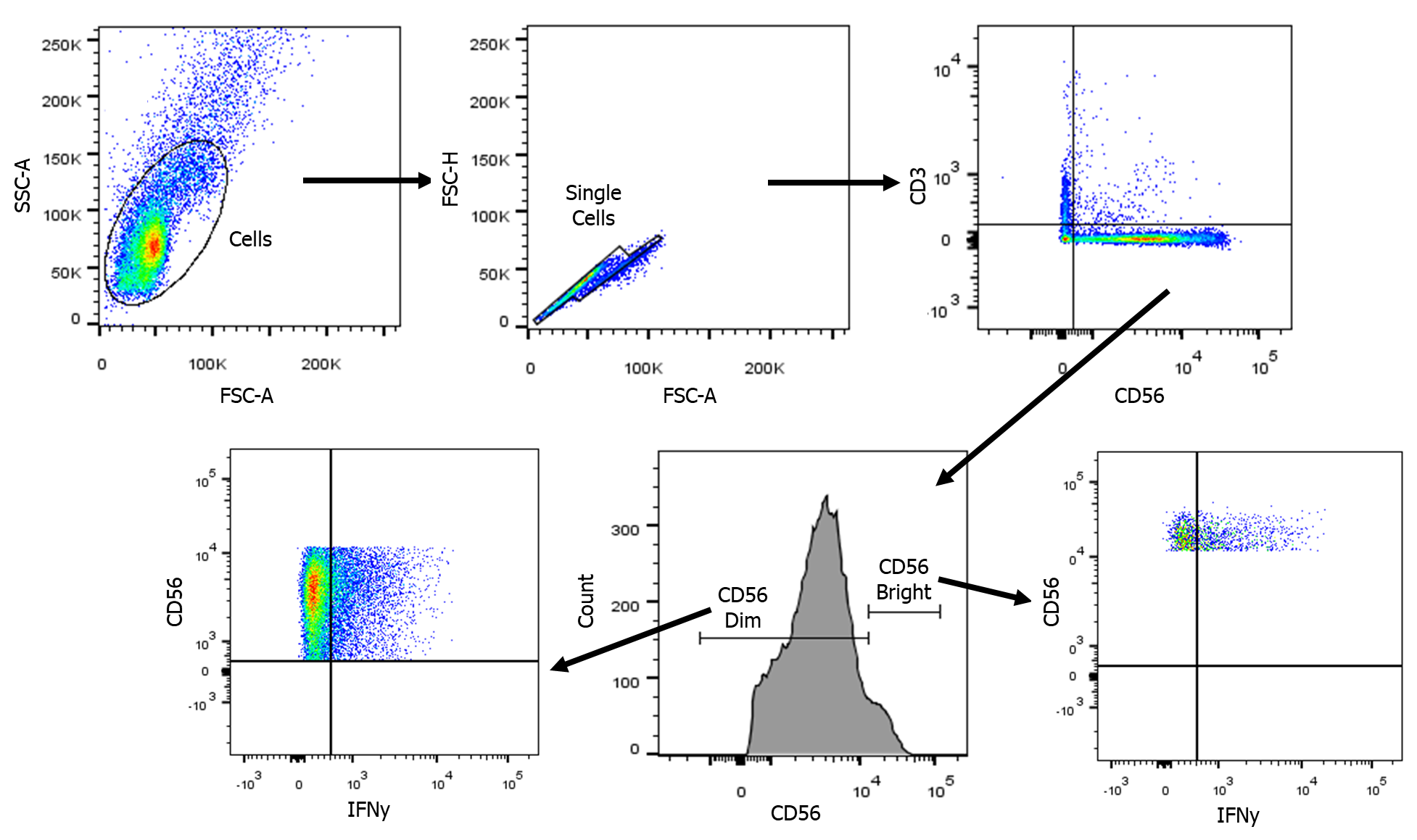
**

**Figure S1.** Gating strategy for stratification of CD56^+^/CD3^-^/IFN-γ^+^ human NK cells.

**(A)**

**
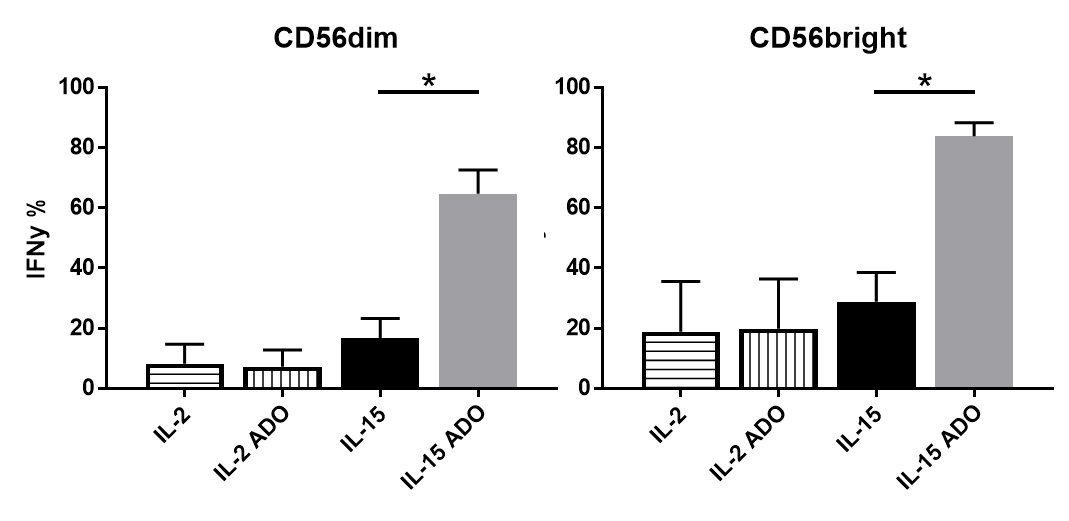
**

**(B)**

**
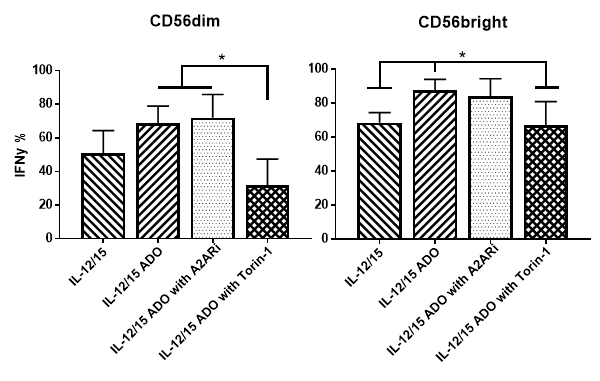
**

**Figure S2. Percentage of IFN-γ^+^ NK cells following activation.** (A) Percentage of IFN-γ^+^ CD56^bright^ and CD56^dim^ subsets following activation of NK cells with IL-2 or IL-15 in the presence or absence of ADO for 24 hours. (Unpaired Student T-test) (B) Percentage of IFN-γ^+^ CD56^bright^ and CD56^dim^ subsets following stimulation of IL-12/IL-15-activated NK cells with or without ADO for 24 hours. (Ordinary one-way ANOVA with Tukey’s multiple comparison) All treatment and analysis conditions are as outlined in Materials and Methods.

**(A)**


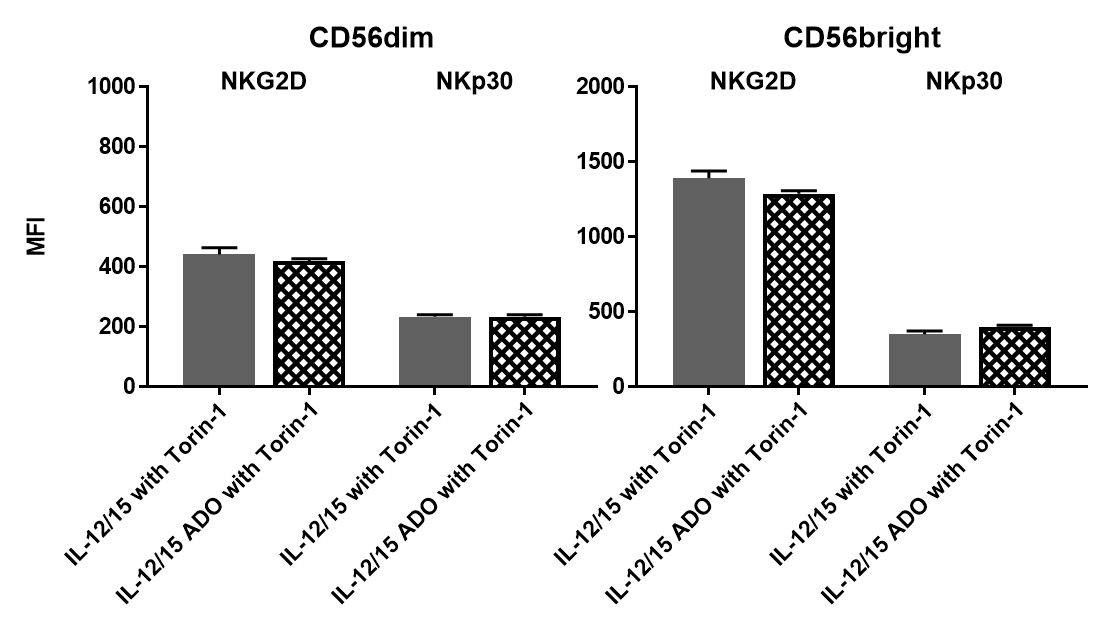


**(B)**

**
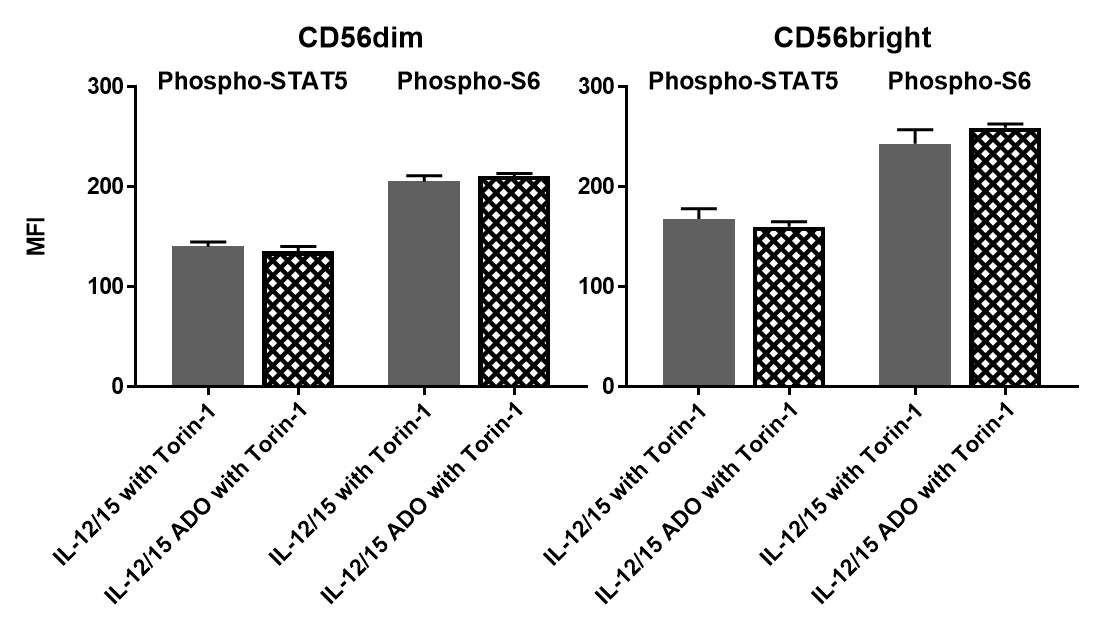
**

**Figure S3. Effect of ADO on NK cells treated with mTOR inhibitor torin-1.** (A) NKG2D and NKp30 expression on freshly-isolated NK cells following incubation with torin-1 (24 h) in the presence or absence of ADO (Unpaired Student t-test); (B) Phospho-STAT5 and Phospho-s6 expression on freshly-isolated NK cells following incubation with torin-1 (24 h) in the presence or absence of ADO (Student t-test).

**(A)**


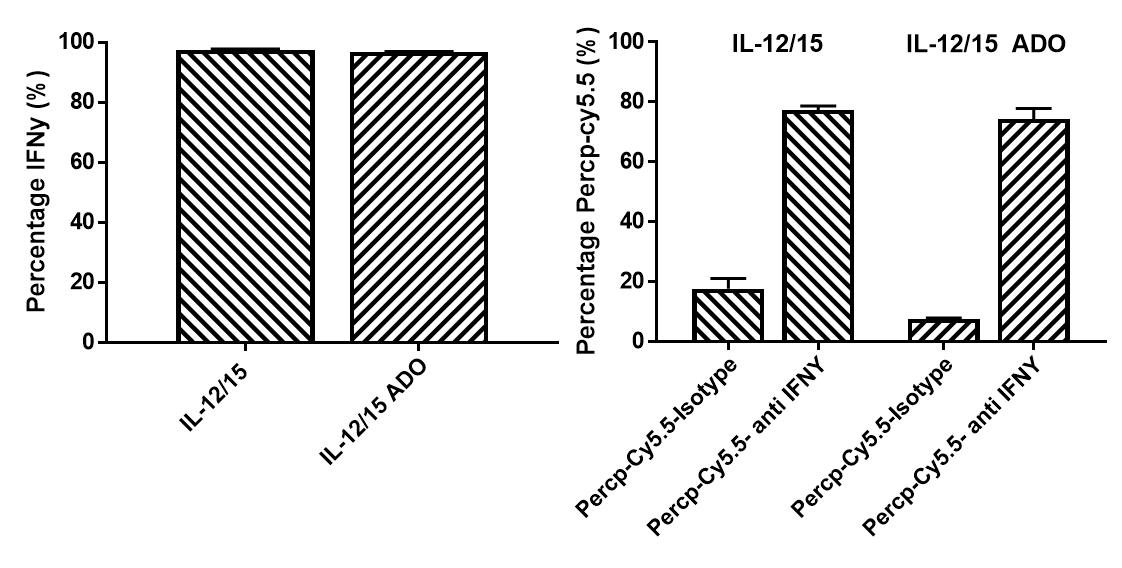


**(B)**


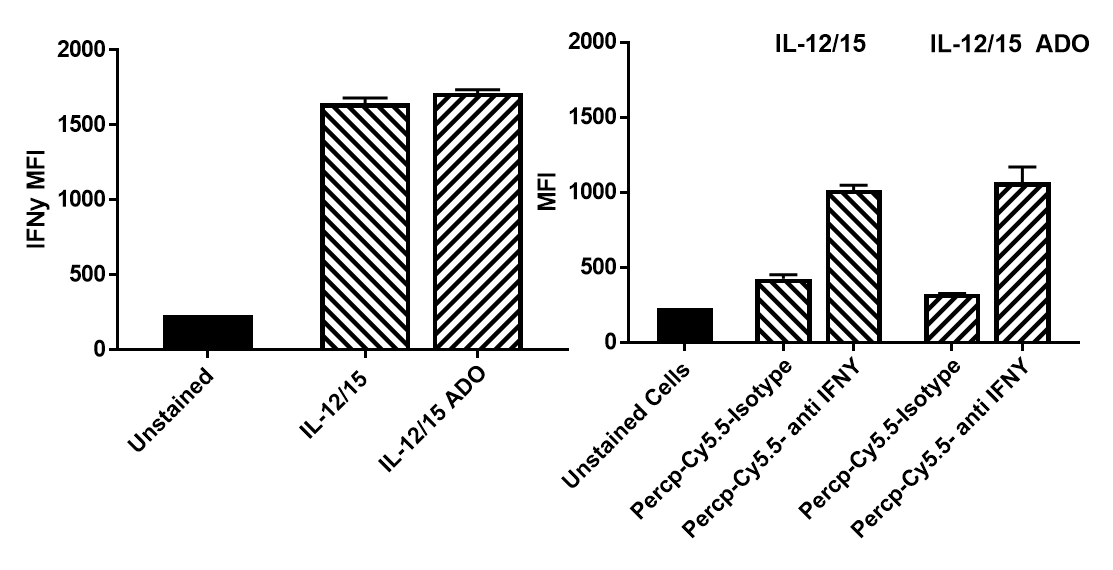


**Figure S4. ADO-mediated effects on IL-12/IL-15-stimulated NK cells with FMO controls and isotype antibody. (A)** *Left panel*, Percentage of IFN-γ^+^ NK cells following stimulation with IL-12/IL-15 in the presence and absence of ADO. *Right panel*, IFN-γ^+^ NK cells stained with Percp-Cy5.5 compared to isotype control. **(B)** *Left panel*, MFI of IFN-γ^+^ NK cells following stimulation with IL-12/IL-15 in the presence and absence of ADO, including unstained sample. *Right panel*, IFN-γ^+^ NK cells **s**tained with Percp-Cy5.5 compared to isotype control including unstained sample.

**(A)**


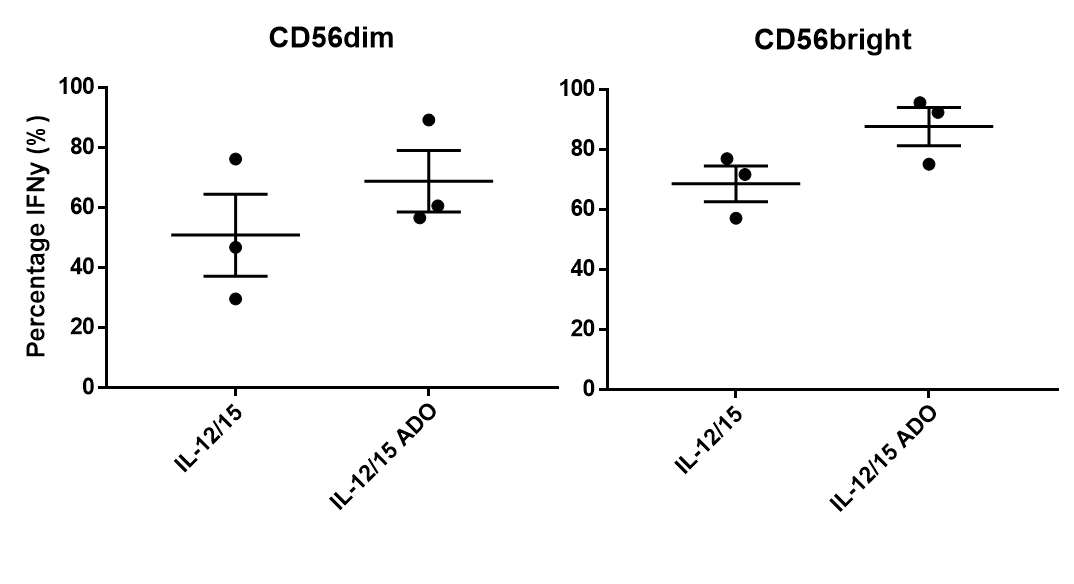


**(B)**

**
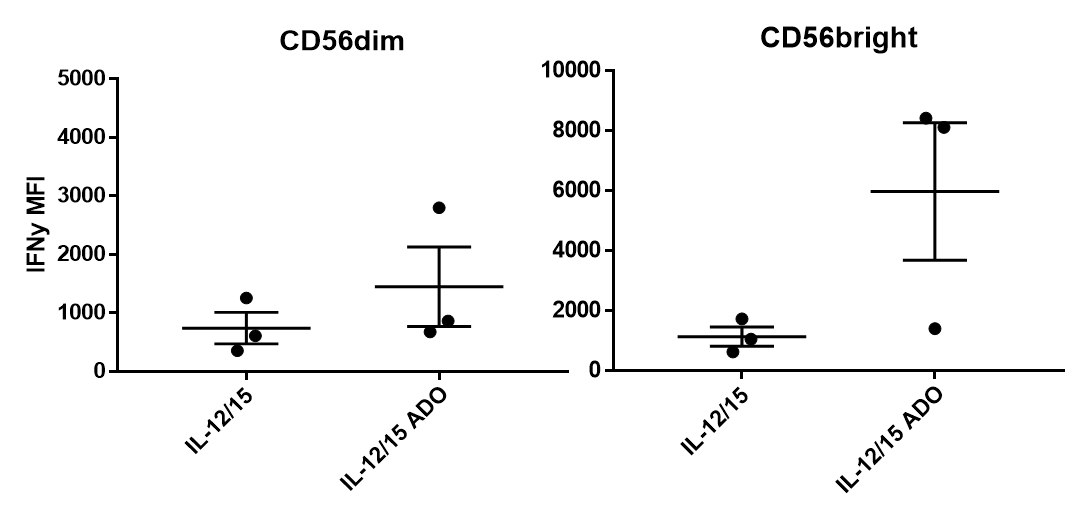
**

**Figure S5**. **Donor distribution.** Representative whisker plots showing individual donors (3 are shown) for (A) percentage IFN-γ^+^ and (B) MFI for IFN-γ-expressing CD56^bright^ and CD56^dim^ NK cells following stimulation with IL-12/IL-15 with or without ADO.


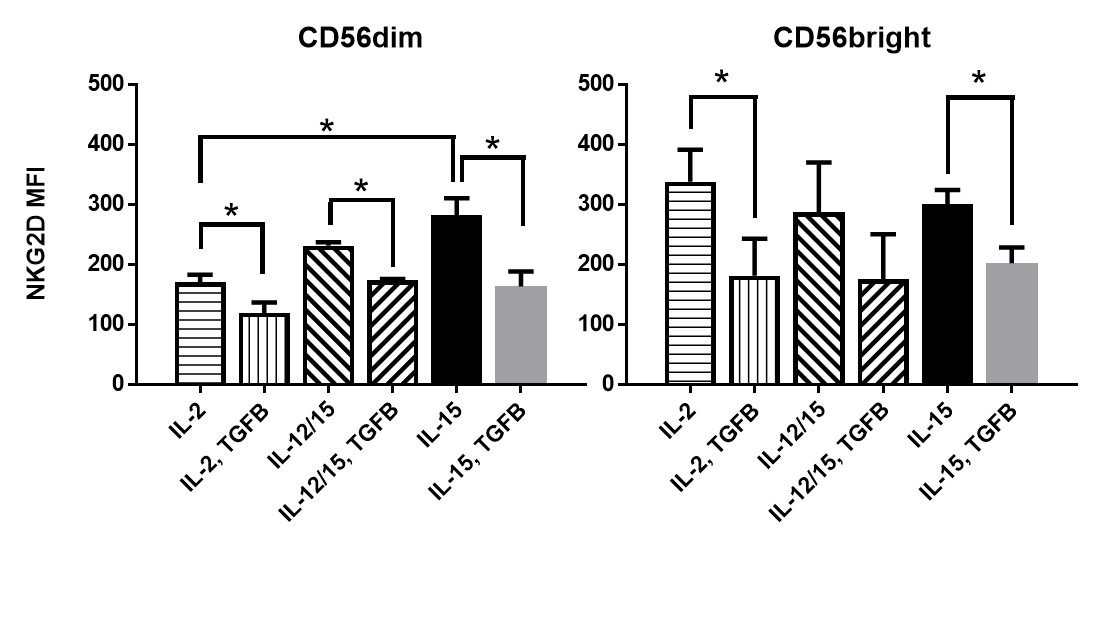


**Figure S6.** Effect of TGF-β on expression of NKG2D. IL-2, IL-15 or IL-12/IL-15-stimulated human NK cells were treated with 10 ng/ml TGF-β for 24 hours prior to analysis of NKG2D expression by flow cytometry Human NK cells were isolated immediately prior to the experiment and stratified as CD56dim and CD56bright (Kruskal-Wallis test with Dunn’s multiple comparison test).


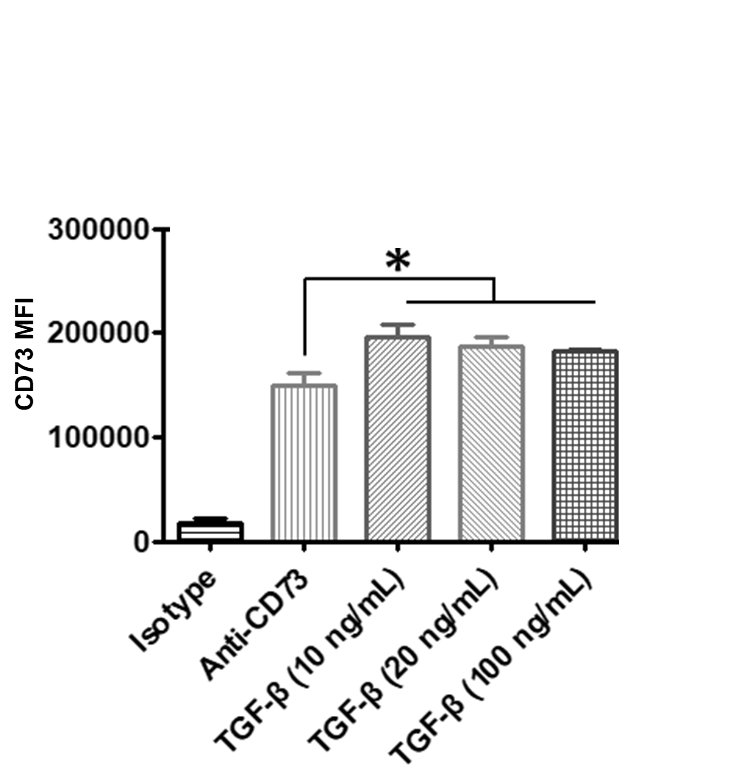


**Figure S7.** **Effect of TGF-β on expression of CD73 on cancer cells.** Stimulation with TGF-β induced a slight increase in the expression of CD73 of A549 cells. A549 cells were stimulated with TGF-β for 24 hours at three concentrations. CD73 expression was measured by flow cytometry with either fluorescently-labeled anti-CD73 antibody or isotype control. The change in expression was not TGF-β concentration dependent (ANOVA).

|  |  |
| --- | --- |
|  |  |

**Figure S8.** **Cytotoxicity of human NK cells against CD73^+^ A549 cells with EHNA and in the presence of ADO.** The addition of 100 µM EHNA mediated a lower cytolysis of A549 target cells by IL-12/IL-15-activated NK cells in the presence of ADO.

1. **(B)**

**Figure S9.** **HLA staining of A549 cells and cytotoxicity of NK cells against K562 cells. (A)** Fold-change of HLA A, B, C expression on A549 cells compared to isotype control. **(B)** Cytotoxicity of human NK cells stimulated with IL-12/IL-15 against K562 cells. Killing was measured over a period of 2 hours, following 24 hours of activation of NK cells with IL-12/IL-15 with or without ADO. Analysis and staining performed as described in Materials and Methods.

**Figure S10.** **Glycolysis stress test on IL-12/IL-15-activated human NK cells.** IL-12 and IL-15-primed NK cells downregulate glycolysis in response to ADO to reach levels close to baseline, while they maintain upregulated glycolytic capacity when primed with IL-12 and IL-15 alone.

**Table S1. Differentially-expressed genes in response to ADO signaling on NK cells.** Complete list of upregulated and downregulated genes on IL-12 and IL-15 co-stimulated human NK cells in response to ADO as determined via DESeq2 and EdgeR analysis.

|  | **DESeq2** | | | | **edgeR** | | | |
| --- | --- | --- | --- | --- | --- | --- | --- | --- |
| **Gene name** | **log2FC** | **lfcSE** | **pvalue** | **padj** | **logFC** | **logCPM** | **PValue** | **FDR** |
| GCFC2 | -0.68503 | 0.197565 | 0.000526 | 0.015344 | -0.69687 | 6.415589 | 9.39E-05 | 0.02925 |
| NOP16 | -0.47465 | 0.108827 | 1.29E-05 | 0.000725 | -0.49072 | 8.272715 | 7.07E-05 | 0.024406 |
| LAG3 | 0.56881 | 0.136403 | 3.05E-05 | 0.001477 | 0.552337 | 7.479216 | 8.68E-05 | 0.027813 |
| TOX4 | -0.48903 | 0.137104 | 0.000361 | 0.011358 | -0.5052 | 7.506521 | 0.000302 | 0.063368 |
| GZMB | 0.460296 | 0.090733 | 3.91E-07 | 3.73E-05 | 0.44342 | 8.793119 | 0.000139 | 0.037782 |
| NFKBIA | 0.640927 | 0.156465 | 4.2E-05 | 0.00182 | 0.62401 | 6.975035 | 6.4E-05 | 0.023159 |
| NPRL3 | -0.50205 | 0.131469 | 0.000134 | 0.00492 | -0.51717 | 7.499213 | 0.000219 | 0.051002 |
| BRAT1 | 0.623577 | 0.122082 | 3.26E-07 | 3.3E-05 | 0.606567 | 7.768151 | 5.78E-06 | 0.002946 |
| PDAP1 | -0.52163 | 0.086316 | 1.51E-09 | 2.06E-07 | -0.53799 | 9.474274 | 9.25E-07 | 0.000569 |
| NFKB1 | 0.685667 | 0.202442 | 0.000707 | 0.019629 | 0.668773 | 6.221984 | 0.0004 | 0.081572 |
| IFNG | 1.412791 | 0.113522 | 1.49E-35 | 2.13E-32 | 1.395132 | 8.384297 | 9.88E-29 | 2.22E-24 |
| GAPDH | -0.47852 | 0.132798 | 0.000314 | 0.010212 | -0.49429 | 7.457004 | 0.000454 | 0.090921 |
| KLC1 | -0.40457 | 0.09702 | 3.05E-05 | 0.001477 | -0.42159 | 8.709153 | 0.000328 | 0.068079 |
| PKMYT1 | -0.64201 | 0.146262 | 1.14E-05 | 0.00065 | -0.6567 | 7.395637 | 4.48E-06 | 0.002395 |
| PHF10 | -0.71006 | 0.155873 | 5.23E-06 | 0.00034 | -0.72501 | 7.026487 | 2.73E-06 | 0.001572 |
| ENOSF1 | -0.63064 | 0.085968 | 2.2E-13 | 5.73E-11 | -0.64762 | 9.057629 | 1.27E-08 | 1.3E-05 |
| ENAM | -0.69667 | 0.083602 | 7.88E-17 | 3.22E-14 | -0.71266 | 10.02874 | 1.76E-11 | 4.39E-08 |
| LDHA | -0.83027 | 0.232445 | 0.000354 | 0.011265 | -0.8452 | 5.773367 | 0.000107 | 0.031923 |
| IL2RA | 0.859343 | 0.209512 | 4.1E-05 | 0.001819 | 0.842179 | 6.064122 | 2.29E-05 | 0.010276 |
| IRF4 | 0.798221 | 0.242389 | 0.000991 | 0.02539 | 0.784911 | 5.940331 | 0.000143 | 0.037782 |
| ADCY3 | -0.48996 | 0.115058 | 2.06E-05 | 0.001091 | -0.5051 | 8.126537 | 5.98E-05 | 0.02236 |
| FAM149B1 | -0.41019 | 0.092352 | 8.93E-06 | 0.000532 | -0.42687 | 9.178203 | 0.000143 | 0.037782 |
| ZNF740 | -0.66312 | 0.065849 | 7.47E-24 | 5.34E-21 | -0.67845 | 10.66302 | 4.29E-11 | 8.74E-08 |
| RMND5B | -0.41522 | 0.100639 | 3.69E-05 | 0.001704 | -0.43114 | 8.737301 | 0.000229 | 0.051463 |
| UBXN11 | -0.39731 | 0.083671 | 2.05E-06 | 0.000147 | -0.41392 | 9.897481 | 9.9E-05 | 0.030072 |
| PAQR4 | -1.05382 | 0.228314 | 3.92E-06 | 0.000267 | -1.06707 | 5.928912 | 3.32E-07 | 0.000254 |
| INTS8 | -0.9242 | 0.257141 | 0.000325 | 0.010463 | -0.93517 | 5.779801 | 1.86E-05 | 0.008671 |
| FAM69B | -0.5678 | 0.172878 | 0.001022 | 0.02539 | -0.58307 | 6.867716 | 0.000263 | 0.057829 |
| SEPT2 | -0.62267 | 0.177915 | 0.000466 | 0.01402 | -0.63802 | 6.617343 | 0.000166 | 0.040442 |
| STARD5 | -0.47102 | 0.118251 | 6.8E-05 | 0.002702 | -0.48761 | 7.949739 | 0.000163 | 0.040442 |
| SNX32 | -0.38662 | 0.078184 | 7.62E-07 | 6.6E-05 | -0.40244 | 11.5754 | 5.3E-05 | 0.020834 |
| TYMSOS | -0.82023 | 0.135544 | 1.44E-09 | 2.05E-07 | -0.83516 | 7.661461 | 1.1E-09 | 1.64E-06 |
| PARP10 | 0.540785 | 0.112378 | 1.49E-06 | 0.000119 | 0.524508 | 8.133639 | 3.07E-05 | 0.01299 |
| AC073072.1 | 2.062861 | 0.239307 | 6.69E-18 | 3.19E-15 | 2.043909 | 6.163449 | 7.53E-23 | 5.63E-19 |
| BCL9L | 0.443013 | 0.084655 | 1.67E-07 | 1.83E-05 | 0.425219 | 9.181831 | 0.000151 | 0.038813 |
| BLM | 0.737116 | 0.220857 | 0.000845 | 0.022601 | 0.719202 | 5.943438 | 0.000473 | 0.093049 |
| MT-ND4 | 0.389866 | 0.061151 | 1.82E-10 | 2.75E-08 | 0.373736 | 11.09161 | 0.000214 | 0.050477 |
| MT-TY | -0.43195 | 0.102851 | 2.67E-05 | 0.001341 | -0.44843 | 8.391037 | 0.000228 | 0.051463 |
| MIF-AS1 | -0.33165 | 0.072686 | 5.05E-06 | 0.000336 | -0.34776 | 11.36515 | 0.00051 | 0.09809 |
| C10orf55 | -0.54032 | 0.109105 | 7.34E-07 | 6.56E-05 | -0.55713 | 8.174939 | 8.52E-06 | 0.004152 |
| SLC25A5-AS1 | -0.44204 | 0.086624 | 3.34E-07 | 3.3E-05 | -0.45862 | 9.514242 | 2.65E-05 | 0.011671 |
| ENTPD1-AS1 | 0.621836 | 0.150245 | 3.49E-05 | 0.00165 | 0.605495 | 7.160494 | 5.54E-05 | 0.021421 |
| AP001610.1 | -0.46972 | 0.134336 | 0.000471 | 0.014044 | -0.48651 | 8.100304 | 0.00012 | 0.03484 |
| AL139289.1 | -0.56362 | 0.158197 | 0.000367 | 0.011412 | -0.57878 | 6.874753 | 0.00028 | 0.060328 |
| AL137186.1 | 0.515174 | 0.075652 | 9.78E-12 | 1.97E-09 | 0.49806 | 9.890602 | 2.9E-06 | 0.001628 |
| AP000688.1 | -0.62062 | 0.171506 | 0.000296 | 0.009897 | -0.63546 | 6.84855 | 7.53E-05 | 0.025216 |
| HLA-DPA1 | -0.39503 | 0.082626 | 1.74E-06 | 0.000131 | -0.41157 | 9.413503 | 0.000183 | 0.044186 |
| CYP1B1-AS1 | -1.36881 | 0.180333 | 3.19E-14 | 1.01E-11 | -1.38313 | 6.759326 | 2.91E-16 | 1.09E-12 |
| AL133415.1 | -0.42655 | 0.063817 | 2.32E-11 | 4.16E-09 | -0.44313 | 10.42144 | 1.89E-05 | 0.008671 |
| ZRANB2-AS1 | -0.45986 | 0.111669 | 3.82E-05 | 0.001735 | -0.47636 | 8.165508 | 0.000142 | 0.037782 |
| OR2I1P | 0.828522 | 0.174035 | 1.93E-06 | 0.000142 | 0.810564 | 6.74675 | 9.39E-07 | 0.000569 |
| AC025164.1 | 0.775432 | 0.182709 | 2.19E-05 | 0.001142 | 0.759135 | 9.819745 | 1.61E-12 | 5.14E-09 |
| OIP5-AS1 | -0.71726 | 0.218637 | 0.001036 | 0.02539 | -0.7322 | 5.966506 | 0.000333 | 0.068605 |
| AC244502.1 | -0.53994 | 0.079384 | 1.03E-11 | 1.97E-09 | -0.55679 | 10.19134 | 1.1E-07 | 9.47E-05 |
| AC009630.2 | -0.6609 | 0.196644 | 0.000777 | 0.021168 | -0.67424 | 6.334986 | 0.000221 | 0.051002 |
| AC007991.4 | -0.82552 | 0.204012 | 5.2E-05 | 0.002221 | -0.83808 | 6.408698 | 3.05E-06 | 0.001669 |
| AC026333.3 | -1.22666 | 0.22876 | 8.22E-08 | 9.41E-06 | -1.24129 | 6.100497 | 5.22E-10 | 9.01E-07 |
| AC011603.2 | -0.46154 | 0.069742 | 3.64E-11 | 6.13E-09 | -0.47754 | 13.54824 | 7.84E-07 | 0.000517 |
| AC125611.3 | -0.52769 | 0.081727 | 1.07E-10 | 1.7E-08 | -0.54456 | 9.84672 | 3.4E-07 | 0.000254 |
| IL21R-AS1 | 0.841824 | 0.117105 | 6.54E-13 | 1.44E-10 | 0.824361 | 8.061486 | 1.32E-10 | 2.47E-07 |
| AC116348.3 | -0.55679 | 0.073812 | 4.58E-14 | 1.31E-11 | -0.57317 | 9.89872 | 7.37E-08 | 6.61E-05 |
| MMP25-AS1 | -1.16648 | 0.089714 | 1.19E-38 | 3.4E-35 | -1.18146 | 9.207321 | 3.19E-25 | 3.57E-21 |
| AC018521.2 | -0.62714 | 0.164098 | 0.000132 | 0.00492 | -0.64235 | 6.834493 | 6.56E-05 | 0.02334 |
| AC084125.4 | -0.88494 | 0.195234 | 5.82E-06 | 0.000366 | -0.89677 | 6.435419 | 5.2E-07 | 0.000376 |
| AC010761.4 | 0.547689 | 0.136202 | 5.79E-05 | 0.002401 | 0.531677 | 7.633624 | 9.92E-05 | 0.030072 |
| AC005786.3 | -0.81209 | 0.14093 | 8.3E-09 | 1.08E-06 | -0.82719 | 7.413761 | 7.78E-09 | 9.18E-06 |
| AC004264.1 | -1.18868 | 0.119111 | 1.87E-23 | 1.07E-20 | -1.20488 | 8.03934 | 2.43E-20 | 1.09E-16 |
| AC006064.4 | -0.58637 | 0.051174 | 2.13E-30 | 2.04E-27 | -0.60236 | 13.13053 | 5.91E-10 | 9.47E-07 |
| AC012073.1 | -0.6717 | 0.186054 | 0.000306 | 0.010061 | -0.68681 | 6.417988 | 0.000117 | 0.034665 |
| AL139423.1 | 0.473385 | 0.086848 | 5.02E-08 | 5.98E-06 | 0.45748 | 9.139148 | 4.88E-05 | 0.019549 |
| AL662797.1 | -0.40764 | 0.056544 | 5.62E-13 | 1.34E-10 | -0.42394 | 12.67517 | 1.42E-05 | 0.006769 |
| AC114271.1 | -0.3598 | 0.070254 | 3.03E-07 | 3.21E-05 | -0.37602 | 10.50958 | 0.000266 | 0.058022 |
| AC093512.1 | -0.60922 | 0.07727 | 3.16E-15 | 1.13E-12 | -0.62561 | 10.4009 | 1.75E-09 | 2.46E-06 |
| PCGF2 | -0.74988 | 0.193967 | 0.000111 | 0.004165 | -0.76431 | 6.301433 | 3.52E-05 | 0.014609 |
| AC010542.5 | -0.64122 | 0.148918 | 1.66E-05 | 0.000915 | -0.65595 | 7.29574 | 6.96E-06 | 0.003466 |
| AC015813.5 | -0.38376 | 0.078033 | 8.75E-07 | 7.36E-05 | -0.40025 | 9.959761 | 0.000158 | 0.039688 |
| AC135048.4 | -0.98245 | 0.204595 | 1.57E-06 | 0.000122 | -0.99772 | 6.249515 | 1.3E-07 | 0.000108 |


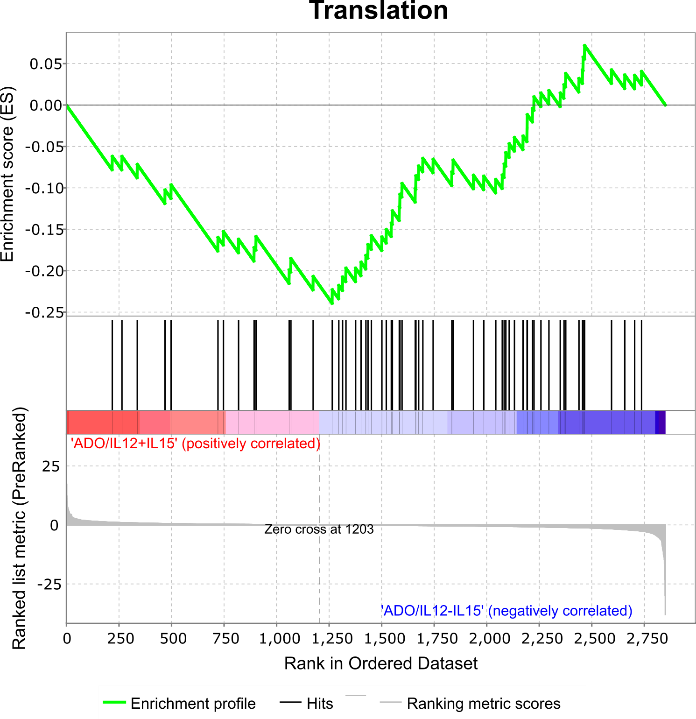

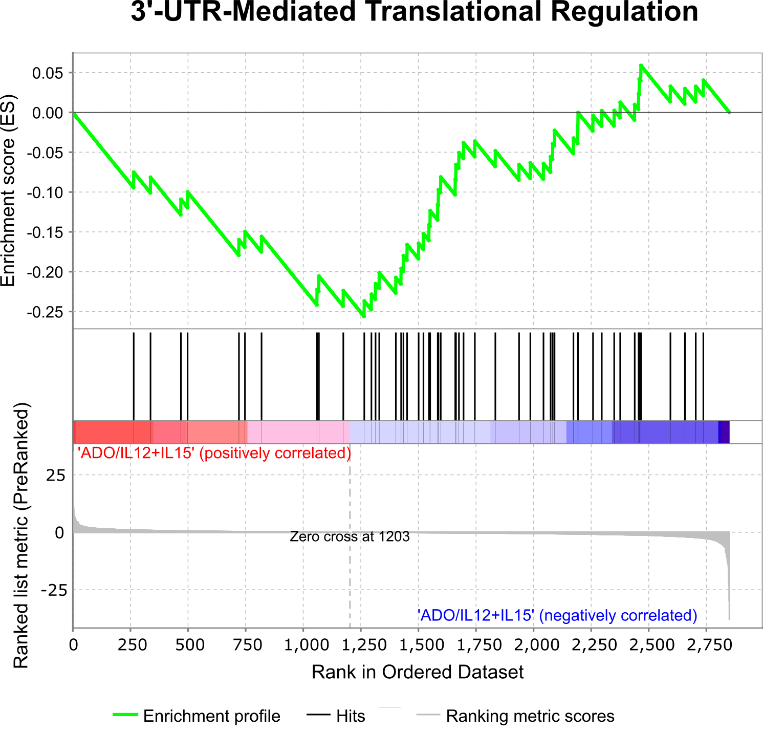


**Figure S11.** **GSEA enrichment plots for downregulated gene sets identified ia Reactome analysis.** Enrichment plots of the two most highly downregulated gene sets on IL-12 and IL-15-primed human NK cells in response to ADO. Gene sets were identified following Reactome database analysis.

**Table S2. GSEA analysis of the top three up and downregulated gene sets in response to ADO signaling on NK cells.** Reactome GSEA analysis of the three most upregulated and downregulated gene sets following treatment of IL-12 and IL-15-primed human NK cells with ADO.

|  | Upregulated Gene Set | SIZE | ES | NES |
| --- | --- | --- | --- | --- |
| 1 | SIGNALING BY ILS | 22 | 0.47 | 2.62 |
| 2 | IMMUNE SYSTEM | 137 | 0.17 | 2.26 |
| 3 | SIGNALING IN IMMUNE SYSTEM | 56 | 0.26 | 2.20 |

|  | Downregulated Gene Set | SIZE | ES | NES |
| --- | --- | --- | --- | --- |
| 1 | TRANSLATION | 64 | -0.24 | -2.24 |
| 2 | 3’-UTR-MEDIATED TRANSLATION REGULATION | 52 | -0.26 | -2.21 |
| 3 | PEPTIDE CHAIN ELONGATION | 45 | -0.27 | -2.08 |
